# Immunogenicity and Safety of the Adjuvanted Respiratory Syncytial Virus Prefusion F Protein-Based Vaccine When Co-administered With the Adjuvanted Recombinant Herpes Zoster Subunit Vaccine in Adults ≥ 50 Years of Age

**DOI:** 10.1093/cid/ciag208

**Published:** 2026-04-29

**Authors:** Jean-François Roussy, Patrick Dennis, Anil K Gupta, Garry Wallace, Alexander Abitbol, Naresh Aggarwal, Joseph G Surber, Theodore Lee, Brian A Smith, Catherine Gérard, Hiwot Amare Hailemariam, Marie-Pierre David, Archana M Jastorff, Marie Van der Wielen

**Affiliations:** DIEX Research, Québec, Québec, Canada; DELRIGHT Research, New Orleans, Louisiana, USA; Albion Finch Medical Center, Toronto, Ontario, Canada; Dawson Clinical Research, Guelph, Ontario, Canada; LMC Healthcare/Centricity Research, Toronto, Ontario, Canada; Aggarwal and Associates, Brampton, Ontario, Canada; Centricity Research Talbotton, Columbus, Georgia, USA; Headlands Research, Orlando, Florida, USA; CCT Research, Versailles, Kentucky, USA; GSK, Rixensart, Belgium; GSK, Wavre, Belgium; GSK, Wavre, Belgium; GSK, Wavre, Belgium; GSK, Wavre, Belgium

**Keywords:** respiratory syncytial virus, herpes zoster, adjuvanted vaccines, safety, co-administration

## Abstract

**Background:**

Co-administration of AS01_E_-adjuvanted respiratory syncytial virus vaccine (adjuvanted RSVPreF3) and AS01_B_-adjuvanted recombinant herpes zoster subunit vaccine (RZV), although not contraindicated, has not been previously studied in clinical trials.

**Methods:**

This phase III, open-label, randomized (1:1) trial evaluated the immunogenicity, safety, and reactogenicity of adjuvanted RSVPreF3 when co-administered with RZV in ≥50-year-olds. The 2 vaccines were either co-administered at visit 1 (day [D] 1; Co-Ad), or were given sequentially, 1 month apart (RZV dose 1 at D1, adjuvanted RSVPreF3 at D31; Control). All participants received RZV dose-2 at D61. Non-inferiority of humoral immune responses was demonstrated if 95% confidence interval (CI) upper limits for geometric mean RSV-A/B neutralizing titers (GMT) and varicella-zoster virus anti-glycoprotein E (anti-gE) antibody concentration (GMC) ratios (Control/Co-Ad) were ≤1.5. Safety and reactogenicity were secondary endpoints.

**Results:**

Overall, 530 participants (Co-Ad = 265; Control = 265) were vaccinated. Neutralizing GMT ratios for RSV-A (1.1 [95% CI = 1.0–1.4]) and RSV-B (1.0 [0.8–1.2]), and anti-gE antibody GMC ratio (1.2 [1.1–1.4]) met non-inferiority criteria. Within 7 days post-vaccination, 71.7% (Co-Ad: post-RZV dose 1 and adjuvanted RSVPreF3; grade [Gr] 3 = 3.9%), 58.5% (Control: post-RZV dose 1; Gr3 = 1.9%), and 47.8% (Control: post-adjuvanted RSVPreF3; Gr3 = 1.2%) of participants reported solicited administration-site adverse events (AEs). Post-visit 1, 73.6% (Gr3 = 9.3%) of Co-Ad and 60.4% (Gr3 = 6.2%) of control participants reported solicited systemic AEs. Within 30 days post-any vaccination, 23.4% (Gr3 = 0.8%) of Co-Ad and 30.2% (Gr3 = 1.1%) of control participants reported unsolicited AEs. No fatal or related serious AEs were reported.

**Conclusions:**

Immunogenicity, reactogenicity, and safety data support the co-administration of adjuvanted RSVPreF3 and RZV.

**Clinical Trials Registration:**

NCT05966090.

## Teaser

Co-administration of AS01_E_-adjuvanted respiratory syncytial virus prefusion F protein-based vaccine and AS01_B_-adjuvanted recombinant herpes zoster subunit vaccine was immunologically non-inferior to their sequential administration and had a clinically acceptable reactogenicity and safety profile.

Adults ≥ 50 years of age (YOA), immunocompromised individuals, and individuals with underlying medical conditions are at increased risk of both severe respiratory syncytial virus (RSV) disease [1–4] and herpes zoster (HZ), also known as shingles [5–7].

Respiratory syncytial virus is a major cause of acute respiratory infections (ARI) [8]. It was estimated that 5.2 million RSV-ARI cases, 470 000 RSV-ARI hospitalizations, and 33 000 in-hospital deaths occurred in 2019 among adults ≥ 60 YOA from high-income countries [9]. Herpes zoster is a painful, typically unilateral dermatomal rash caused by reactivation of varicella-zoster virus (VZV) in the host [5]. Prior to the introduction of the HZ vaccine, it was estimated that approximately 1 000 000 cases of HZ occurred in the United States annually [10]. One-third of unvaccinated individuals developed HZ in their lifetime [10, 11]. Age is an important risk factor for HZ, with a steep increase in HZ incidence in individuals ≥ 50 YOA [10].

The AS01_E_-adjuvanted RSV prefusion F protein-based vaccine (adjuvanted RSVPreF3, *Arexvy*, GSK) is licensed in the United States, the European Union, and in many countries worldwide for the prevention of RSV lower respiratory tract disease in individuals ≥ 60 YOA and individuals 50–59 YOA at increased risk for severe RSV disease [12–15]. Vaccine recommendations vary among countries where it is licensed [16, 17]. In the United States, the Centers for Disease Control's Advisory Committee on Immunization Practices (ACIP) recommends a single dose of any RSV vaccine for adults ≥ 75 YOA and for adults 50–74 YOA at increased risk for severe RSV disease [18, 19].

The AS01_B_-adjuvanted recombinant HZ subunit vaccine (RZV, *Shingrix*, GSK) is licensed in many regions worldwide for the prevention of HZ in adults ≥ 50 YOA and ≥18 YOA at increased risk of HZ [20–22]. In the United States, ACIP recommends 2 RZV doses for the prevention of HZ in immunocompetent adults ≥ 50 YOA and in adults ≥ 19 YOA who are or will be at increased risk of HZ due to immunodeficiency/immunosuppression caused by known disease or therapy [23].

Adjuvanted RSVPreF3 can be co-administered with other adult vaccines such as the quadrivalent seasonal inactivated influenza vaccines [24–26]. Respiratory syncytial virus can also be co-administered with other routine adult vaccines [27]. This study, in addition to informing on co-administration of 2 vaccines recommended to individuals in the same age group, also provides insights on the co-administration of 2 AS01-adjuvanted vaccines, which, although not contraindicated, has not been previously studied in clinical trials. In this study, we assessed the immunogenicity, safety, and reactogenicity of adjuvanted RSVPreF3 when co-administered with the first RZV dose in adults ≥ 50 YOA as compared to the sequential administration of these 2 vaccines. A plain language summary is provided in Figure 1.

**Figure 1.**
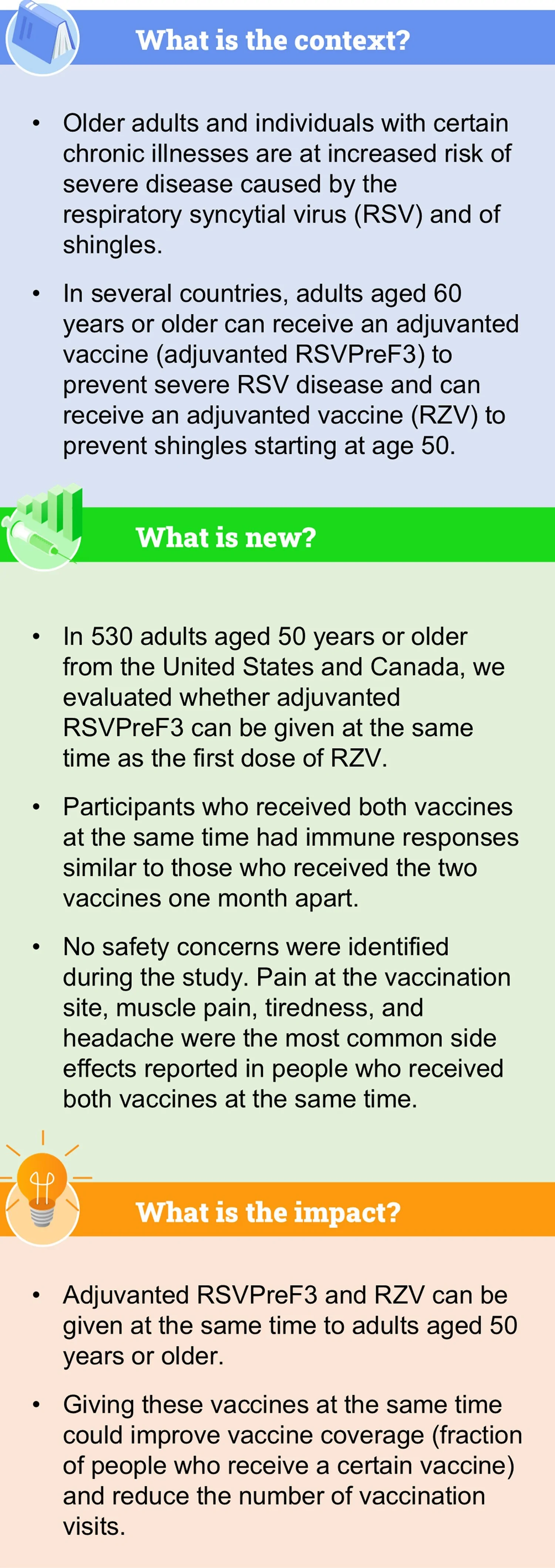
Plain language summary.

## METHODS

### Study Design and Participants

This phase III, open-label, randomized trial was conducted from 28 July 2023 to 29 July 2024, at 20 centers in Canada and the United States. Participants were eligible if they were ≥50 YOA and medically stable (including stable chronic medical conditions) at enrollment. Individuals who were previously vaccinated with any licensed or investigational RSV or HZ vaccine were excluded. Enrollment criteria are detailed in Supplementary methods.

Participants were randomized 1:1 using an internet-based centralized randomization system (Interactive Web Response System). The randomization algorithm used a minimization procedure accounting for age (50–59/60–69/≥70 YOA) and study center; minimization factors had equal weight. Vaccine administration was staggered. A sentinel group comprising 10% of enrolled participants was first vaccinated on D1 and followed for 7 days. Randomization was paused while a safety review team assessed the cumulative safety data. If no safety concerns arose, randomization resumed for the remaining participants.

Participants either received the adjuvanted RSVPreF3 dose concomitantly with the first RZV dose on day (D) 1 (Co-Ad group) or received the first RZV dose on D1 followed by the adjuvanted RSVPreF3 dose on D31 (Control group) (Figure 2). For both groups, a second RZV dose was administered on D61. During an 8-month (M) period, participants attended four site visits (on D1, D31, D61, D91), plus a remote safety contact 6-M post-last vaccination.

**Figure 2.**
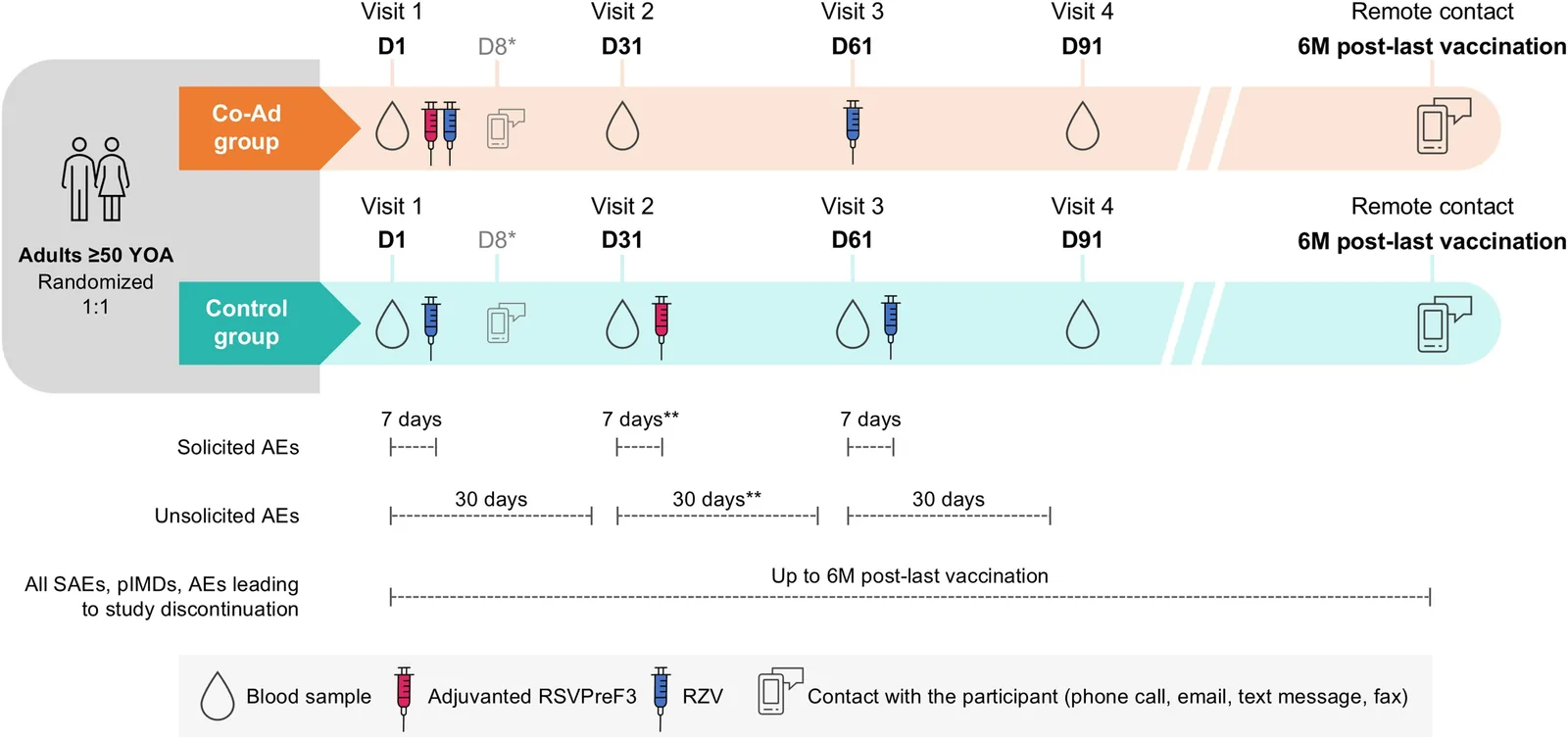
Study design. *A remote contact was conducted on D8 for a sentinel group comprising 10% of the enrolled participants who had received visit 1 vaccinations. Randomization was subsequently paused. A safety review team reviewed the cumulative safety data from the 7 d following visit 1 before resuming randomization for the remaining 90% of participants. **Only for control group. All SAEs and pIMDs, including related SAEs and pIMDs, were recorded up to 6-M post-last vaccination (ie, study end). Abbreviations: adjuvanted RSVPreF3, adjuvanted respiratory syncytial virus prefusion F protein-based vaccine; AEs, adverse events; Co-Ad group, co-administration of RZV dose 1 with adjuvanted RSVPreF3; Control group, sequential administration of RZV dose 1 and adjuvanted RSVPreF3; D, day; M, month; pIMDs, potential immune-mediated diseases; RZV, adjuvanted recombinant herpes zoster subunit vaccine; SAEs, serious AEs; YOA, years of age.

Both participants and principal investigator were aware of the intervention administered. The laboratory performing sample testing was blinded to study interventions. The 2 vaccines were injected in the deltoid muscle of opposite arms when co-administered, or in the non-dominant arm when administered sequentially. Information on vaccine composition is provided in Supplementary methods.

Written or witnessed informed consent was obtained from all participants at enrollment. The study was conducted in accordance with the Declaration of Helsinki, Council for International Organizations of Medical Sciences, the principles of Good Clinical Practice, and all applicable regulatory requirements. The study protocol (available at https://www.gsk-studyregister.com/en/trial-details/?id=219331) was reviewed and approved by Independent Ethics Committees or Institutional Review Boards before study start. The trial is registered at www.clinicaltrials.gov (NCT05966090).

### Objectives

The primary objectives were to demonstrate non-inferiority of the humoral immune responses induced following the co-administration of adjuvanted RSVPreF3 and RZV, compared to their sequential administration, in terms of RSV-A/B neutralizing titers, 1-M post-adjuvanted RSVPreF3 dose, and VZV anti-glycoprotein E (anti-gE) antibody concentration, 1-M post-second RZV dose.

Secondary objectives included further evaluation of the humoral immune responses to both vaccines, in terms of RSV-A/B neutralizing geometric mean titers (GMTs) and mean geometric increases (MGIs), and VZV anti-gE seropositivity rates, geometric mean concentrations (GMCs), MGIs, and vaccine response rates (VRRs, percentage of participants with a 4-fold increase in VZV anti-gE antibody concentration post-vaccination vs pre-vaccination for seropositive participants at baseline, or a 4-fold increase in VZV anti-gE antibody concentration post-vaccination vs the VZV anti-gE antibody cutoff value of 97 milli-international units [IU]/mL for seronegative participants at baseline).

Secondary objectives also included the evaluation of reactogenicity and safety following co-administration versus sequential administration of the 2 vaccines.

### Immunogenicity Assessments

Blood samples (∼10 mL each) were collected from all participants in the Co-Ad group on D1, D31, and D91 and from all participants in the control group on D1, D31, D61, and D91. RSV-A/B neutralizing titers were measured before adjuvanted RSVPreF3 administration (Co-Ad: D1; control: D31) and 1-M post-adjuvanted RSVPreF3 administration (Co-ad: D31; control: D61). Varicella-zoster virus anti-gE antibody concentrations were measured before the first RZV dose (D1) and 1-M post-second RZV dose (D91) using an enzyme-linked immunosorbent assay. Assays and their cutoffs are described in Supplementary methods.

### Safety Assessments

Solicited administration-site adverse events (AEs) (erythema, injection-site pain, swelling) and solicited systemic AEs (arthralgia, fatigue, fever [≥38.0°C/100.4 °F], headache, myalgia, chills [including shivering], gastrointestinal symptoms [nausea, vomiting, diarrhea, and/or abdominal pain]) occurring within 7 days post-any vaccine administration were recorded in electronic diaries (eDiaries). Unsolicited AEs occurring within 30 days post-each vaccine dose were collected during monthly interviews (D1/D31/D61/D91) or at the next visit/contact in the individual electronic case report form. All serious AEs (SAEs), potential immune-mediated diseases (pIMDs), and AEs leading to study discontinuation were recorded in individual electronic case report forms, from D1 until 6 M post-last vaccination (ie, study end).

### Statistical Methods

The study planned to enroll approximately 530 participants to achieve 225 evaluable participants in each group for the analysis of the primary objectives, assuming a 15% drop-out rate. Enrollment rules are shown in Supplementary methods. Participants who withdrew from the study were not replaced. Immunogenicity was assessed in the per-protocol sets (PPS) for RSV or HZ analysis (ie, all eligible participants who received adjuvanted RSVPreF3 [RSV analysis] or 2 RZV doses [HZ analysis] as per protocol in the control group and received all vaccine doses in the Co-Ad group, had available immunogenicity results pre- and post-vaccination, complied with blood draw intervals, had no intercurrent medical conditions that could interfere with immunogenicity, and did not receive prohibited concomitant medication/vaccination). Safety and reactogenicity were assessed in the exposed set (ES; all participants who received ≥1 study intervention).

Each co-primary objective was evaluated with a nominal type I error of 2.5%. Non-inferiority was first evaluated for RSV-A neutralizing titers and VZV anti-gE antibodies and was demonstrated if the upper limits (ULs) of the 2-sided 95% confidence intervals (CIs) for the adjusted group GMT and GMC ratios (Control/Co-Ad) were ≤1.5. If successful, non-inferiority was subsequently evaluated for RSV-B neutralizing titers and was demonstrated if the UL of the 2-sided 95% CI for the adjusted group GMT ratio (control/Co-Ad) was ≤1.5. The least square point estimates and their 2-sided 95% CIs for adjusted group GMT or GMC ratios (control/Co-Ad) were derived from an analysis of covariance model that included treatment group and age category as fixed effects, and pre-vaccination log_10_-transformed titers or concentration as covariates. Missing data were not replaced. Titers or concentrations below the assay cutoffs were replaced by half the assay cutoffs; titers above the ULs of quantification were replaced by the ULs of quantification.

All secondary objectives were analyzed descriptively. The humoral immune response to adjuvanted RSVPreF3 was assessed in terms of RSV-A/B neutralizing GMTs and MGIs. The humoral immune response to RZV was assessed in terms of seropositivity rates (percentage of participants with VZV anti-gE concentration ≥ 97 mIU/mL), GMCs and MGIs, and VRR with exact 95% CIs. Mean geometric increases were calculated as the geometric means of the within-participant ratios of the post-vaccination over the pre-vaccination titers/concentrations. The serological response rate (SRR) with exact 95% CI was calculated in post hoc analyses to assess the percentage of participants with a ≥4-fold increase in neutralizing titers from pre-vaccination to 1-M post-adjuvanted RSVPreF3.

Percentages of participants with solicited or unsolicited AEs, SAEs, and pIMDs were calculated with exact 95% CIs. A sensitivity analysis of solicited AEs was performed for participants who completed all eDiary entries at D1–3, D3–6, and D1–7. Age-stratified subgroup analyses (50–59/60–69/≥70 YOA) were performed both for immunogenicity and safety endpoints; however, these exploratory analyses were not powered for detecting age group differences.

All analyses were conducted using SAS v9.4 or above.

## RESULTS

### Study Participants

In total, 530 participants (265 in each group) were enrolled and vaccinated, of which 511 (96.4%) completed the study, with loss to follow-up as the main reason for discontinuation. The PPS included 240 participants in the Co-Ad group and 219 participants in the control group for the RSV analysis and 198 participants in the Co-Ad group and 170 participants in the control group for the HZ analysis (Figure 3).

**Figure 3.**
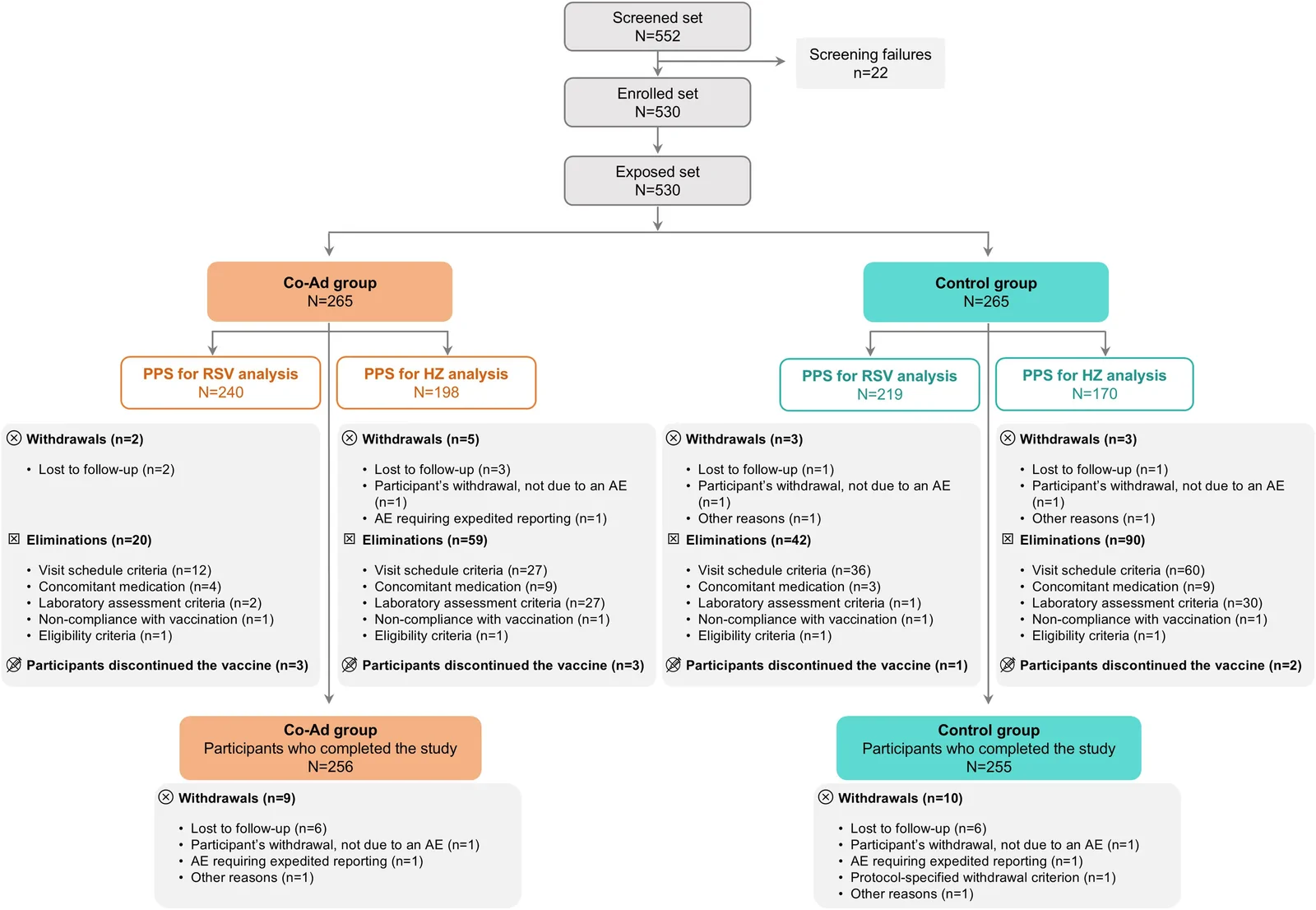
Participant flowchart. The PPS for RSV analysis included all eligible participants who received the adjuvanted RSVPreF3 dose as per protocol in the control group and all eligible participants who received all 3 vaccine doses in the Co-Ad group. The PPS for HZ analysis included all eligible participants who received 2 doses of RZV as per protocol in the control group and all eligible participants who received all 3 vaccine doses in the Co-Ad group. Abbreviations: adjuvanted RSVPreF3, adjuvanted respiratory syncytial virus prefusion F protein-based vaccine; AE, adverse event; Co-Ad group, co-administration of RZV dose 1 with adjuvanted RSVPreF3; Control group, sequential administration of RZV dose 1 and adjuvanted RSVPreF3; HZ, herpes zoster; n, number of participants withdrawn/eliminated; N, number of participants per group/analysis set; PPS, per-protocol set; RSV, respiratory syncytial virus; RZV, adjuvanted recombinant herpes zoster subunit vaccine.

Baseline demographic characteristics were balanced between study groups in the ES and PPS (Table 1, Supplementary Table 1). Participants in the ES had a mean age of 63.9 years. There were slightly more female (55.1%) than male participants (44.9%). Most participants resided in Canada (64.2%) and were White (67.5%). Preexisting medical conditions were reported in 247 (93.2%) participants at enrollment in each group; the types of conditions were balanced between Co-Ad and control groups (Table 1).

**Table 1.** Baseline Characteristics of the Participants (Exposed Set)

Characteristic	Co-Ad Group N = 265	Control Group N = 265	Total N = 530

Age at first dose, mean (SD), years	63.9 (8.6)	63.9 (8.7)	63.9 (8.6)
Age at first dose, median (min–max), years	64.0 (50–87)	63.0 (50–97)	63.0 (50–97)
Age group, n (%)			
50–59 y	92 (34.7)	94 (35.5)	186 (35.1)
60–69 y	93 (35.1)	93 (35.1)	186 (35.1)
≥70 y	80 (30.2)	78 (29.4)	158 (29.8)
≥75 y	34 (12.8)	26 (9.8)	60 (11.3)
Sex, n (%)			
Male	119 (44.9)	119 (44.9)	238 (44.9)
Female	146 (55.1)	146 (55.1)	292 (55.1)
Country, n (%)			
Canada	171 (64.5)	169 (63.8)	340 (64.2)
US	94 (35.5)	96 (36.2)	190 (35.8)
Race, n (%)			
Black or African American	45 (17.0)	45 (17.0)	90 (17.0)
White	178 (67.2)	180 (67.9)	358 (67.5)
Other^a^	42 (15.8)	40 (15.1)	82 (15.5)
Hispanic or Latino ethnicity, n (%)	28 (10.6)	30 (11.3)	58 (10.9)
Preexisting conditions, n (%)			
Any preexisting condition	247 (93.2)	247 (93.2)	494 (93.2)
Metabolism and nutrition disorders	135 (50.9)	136 (51.3)	271 (51.1)
Vascular disorders	131 (49.4)	127 (47.9)	258 (48.7)
Musculoskeletal and connective tissue disorders	106 (40.0)	90 (34.0)	196 (37.0)
Gastrointestinal disorders	91 (34.3)	73 (27.5)	164 (30.9)
Surgical and medical procedures	82 (30.9)	80 (30.2)	162 (30.6)
Social circumstances	73 (27.5)	53 (20.0)	126 (23.8)
Psychiatric disorders	71 (26.8)	69 (26.0)	140 (26.4)
Reproductive system and breast disorders	52 (19.6)	54 (20.4)	106 (20.0)
Nervous system disorders	48 (18.1)	42 (15.8)	90 (17.0)
Respiratory, thoracic, and mediastinal disorders	46 (17.4)	52 (19.6)	98 (18.5)
Immune system disorders	45 (17.0)	32 (12.1)	77 (14.5)
Infections and infestations	37 (14.0)	26 (9.8)	63 (11.9)
Neoplasms benign, malignant, and unspecified (incl. cysts and polyps)	34 (12.8)	30 (11.3)	64 (12.1)
Eye disorders	32 (12.1)	33 (12.5)	65 (12.3)
Renal and urinary disorders	28 (10.6)	26 (9.8)	54 (10.2)
Endocrine disorders	26 (9.8)	28 (10.6)	54 (10.2)
Cardiac disorders	25 (9.4)	28 (10.6)	53 (10.0)
Hepatobiliary disorders	23 (8.7)	24 (9.1)	47 (8.9)
Skin and subcutaneous tissue disorders	23 (8.7)	22 (8.3)	45 (8.5)
Ear and labyrinth disorders	10 (3.8)	9 (3.4)	19 (3.6)
General disorders and administration-site conditions	10 (3.8)	7 (2.6)	17 (3.2)
Blood and lymphatic system disorders	9 (3.4)	10 (3.8)	19 (3.6)
Injury, poisoning, and procedural complications	8 (3.0)	18 (6.8)	26 (4.9)
Investigations	7 (2.6)	14 (5.3)	21 (4.0)
Congenital, familial, and genetic disorders	6 (2.3)	7 (2.6)	13 (2.5)
Pregnancy, puerperium, and perinatal conditions^b^	1 (0.4)	0 (0.0)	1 (0.2)

Abbreviations: adjuvanted RSVPreF3, adjuvanted respiratory syncytial virus prefusion F protein-based vaccine; Co-Ad group, co-administration of RZV dose 1 with adjuvanted RSVPreF3; Control group, sequential administration of RZV dose 1 and adjuvanted RSVPreF3; n (%), number (percentage) of participants in each group with the specified characteristic; N, total number of participants; RZV, adjuvanted recombinant herpes zoster subunit vaccine; SD, standard deviation; US, United States.

^a^Includes Asian, American Indian or Alaska Native, Native Hawaiian or Other Pacific Islanders, multiple, other, or missing.

^b^One participant in the Co-Ad group reported a history of ectopic pregnancy.

### Immunogenicity

The primary objectives of the study were met as the ULs of the 95% CIs of RSV-A/B neutralizing adjusted GMT ratios and the VZV anti-gE antibody adjusted GMC ratio were all ≤1.5 (Figure 4). At 1-M post-adjuvanted RSVPreF3 dose (Co-Ad: D31; control: D61), RSV-A and RSV-B adjusted GMT ratios were 1.1 (1.0–1.4) and 1.0 (0.8–1.2), respectively. At 1-M post-second RZV dose (D91), the adjusted VZV anti-gE antibody GMC ratio was 1.2 (1.1–1.4). Results of age-stratified immunogenicity analyses are shown in Supplementary Table 2.

**Figure 4.**
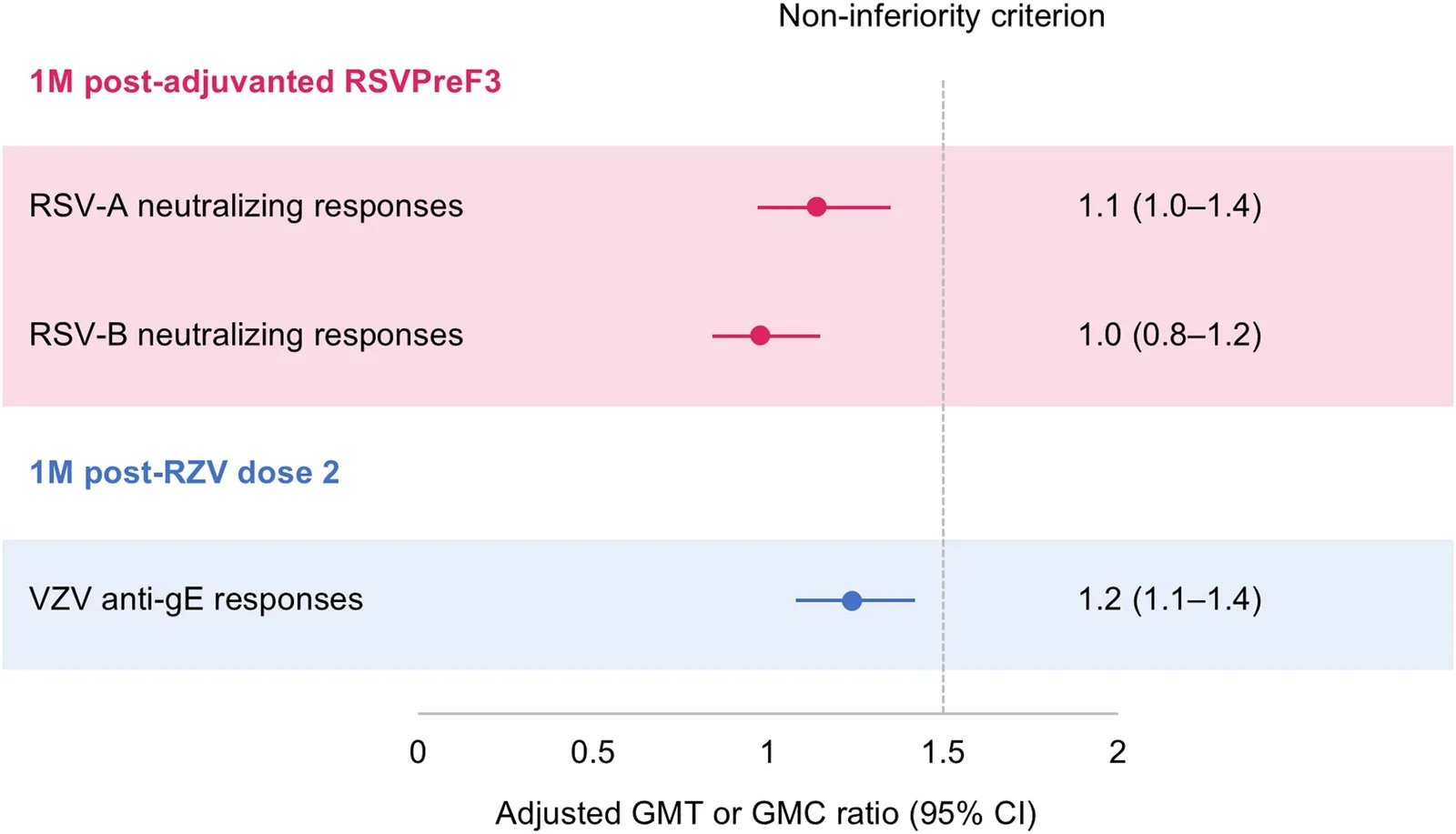
Non-inferiority of adjuvanted RSVPreF3 and RZV co-administration versus sequential administration (per-protocol set). Error bars depict 95% CIs. Success criterion for non-inferiority: upper limits of the 2-sided 95% CIs for group GMT ratios (control/Co-Ad) for each RSV subtype and for group GMC ratio (control/Co-Ad) for VZV anti-gE antibodies were ≤1.5 at 1-M post-vaccination. Abbreviations: adjuvanted RSVPreF3, adjuvanted respiratory syncytial virus prefusion F protein-based vaccine; CI, confidence interval; gE, glycoprotein E; GMC, geometric mean concentration; GMT, geometric mean titer; M, month; RSV-A/B, respiratory syncytial virus subtype A/B; RZV, adjuvanted recombinant herpes zoster subunit vaccine; VZV, varicella-zoster virus.

RSV-A/B neutralizing titers increased from baseline to 1-M post-adjuvanted RSVPreF3 dose in both groups, with MGIs of 8.6 (7.4–9.8) for RSV-A and 9.0 (7.9–10.2) for RSV-B in the Co-Ad group and 9.9 (8.6–11.5) for RSV-A and 8.7 (7.5–10.1) for RSV-B in the control group (Figure 5). Serological response rates were 78.5% (72.9–83.5) for RSV-A and 80.6% (75.1–85.3) for RSV-B in the Co-Ad group and 80.4% (74.5–85.4) for RSV-A and 74.9% (68.6–80.5) for RSV-B in the Control group.

**Figure 5.**
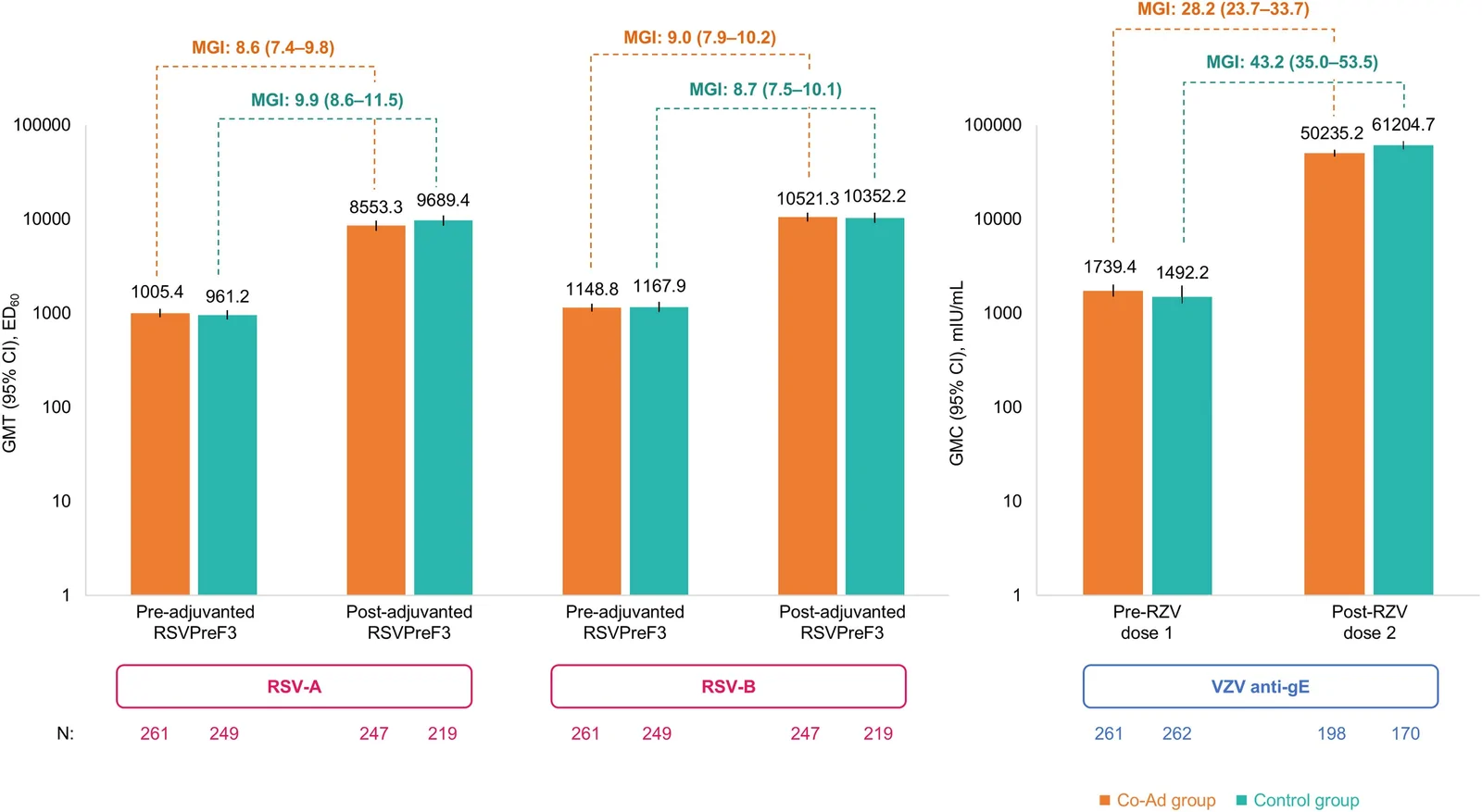
RSV-A and RSV-B neutralizing titers and VZV anti-gE antibody concentrations, at baseline and 1-M post-adjuvanted RSVPreF3 and RZV dose 2 administration (per-protocol set). Abbreviations: adjuvanted RSVPreF3, adjuvanted respiratory syncytial virus prefusion F protein-based vaccine; CI, confidence interval; Co-Ad group, co-administration of RZV dose 1 with adjuvanted RSVPreF3; Control group, sequential administration of RZV dose 1 and adjuvanted RSVPreF3; ED_60_, estimated dilution 60; gE, glycoprotein E; GMC, geometric mean concentration; GMT, geometric mean titer; IU, international units; MGI, mean geometric increase; N, total number of participants; RSV-A/B, respiratory syncytial virus subtype A/B; RZV, adjuvanted recombinant herpes zoster subunit vaccine; VZV, varicella-zoster virus.

Varicella-zoster virus anti-gE antibody concentrations increased from baseline to 1-M post-second RZV dose in both groups, with MGIs of 28.2 (23.7–33.7) in the Co-Ad group and 43.2 (35.0–53.5) in the Control group (Figure 5); all participants in both groups were seropositive 1-M post-second RZV dose. Vaccine response rates in terms of VZV anti-gE antibody concentrations were 92.9% (88.4–96.1) in the Co-Ad group and 96.5% (92.5–98.7) in the Control group.

### Safety and Reactogenicity

Compliance in completing eDiaries was comparable across study groups (Supplementary Table 3). The lowest compliance (60.5%) was observed at Visit 2 for the Control group.

Overall, 77.7% (grade [Gr] 3: 4.2%) of participants in the Co-Ad group and 72.7% (Gr3: 3.8%) of participants in the Control group reported ≥1 solicited administration-site AE occurring within 7 days post-any vaccination. Solicited administration-site AEs were reported for 71.7% (Gr3: 3.9%) of participants within 7 days after co-administration of the 2 vaccines (Co-Ad group) and for 58.5% (Gr3: 1.9%) and 47.8% (Gr3: 1.2%) of participants within 7 days after separate administration of adjuvanted RSVPreF3 and first RZV dose (control group), respectively. Injection-site pain was the most frequent solicited administration-site AE in both groups, for each vaccine (Figure 6*A*; Supplementary Table 4). The median duration of each solicited administration-site AE was 2 days.

**Figure 6.**
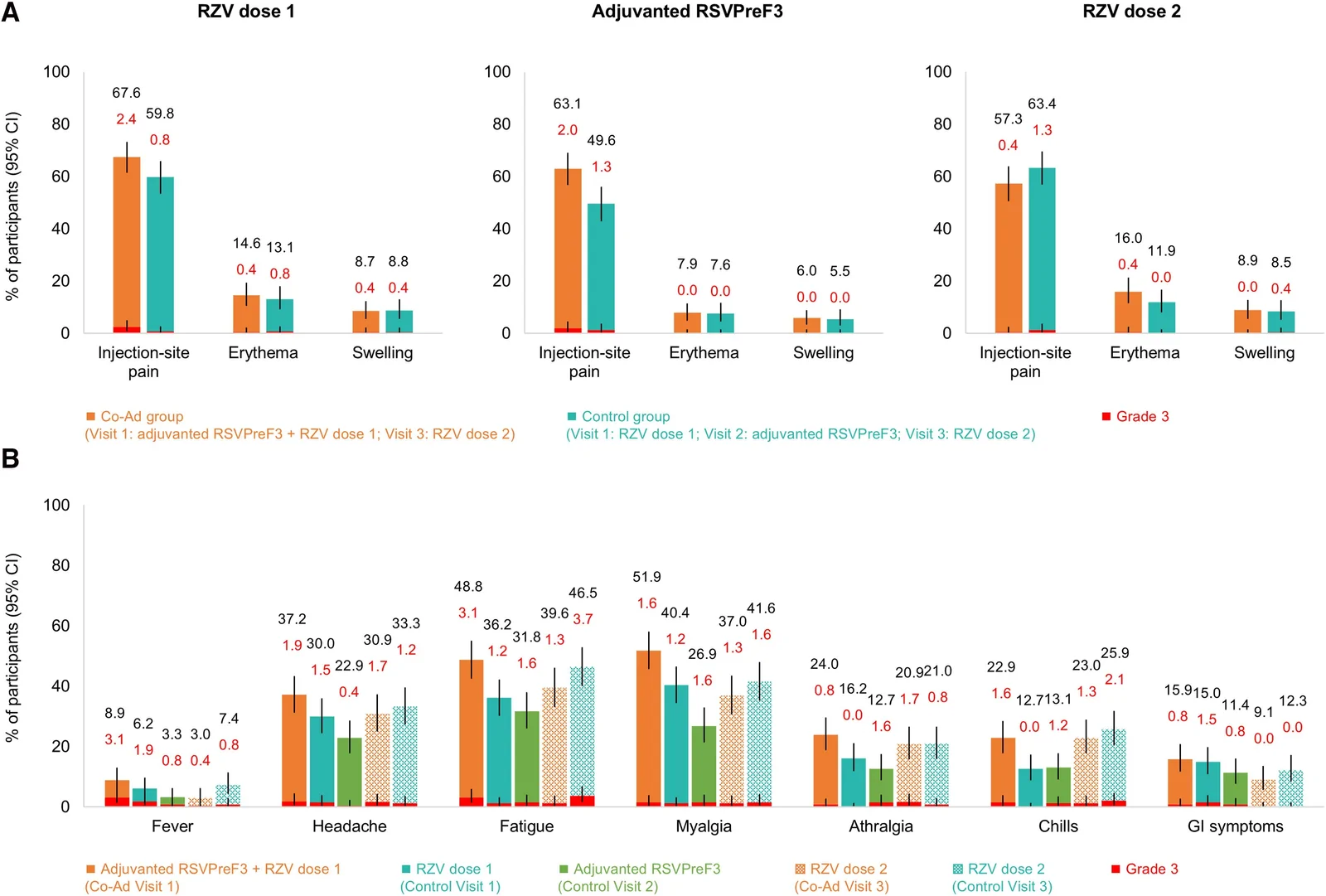
Solicited administration-site (*A*) and systemic (*B*) adverse events (exposed set). Fever was defined as a temperature ≥ 38.0°C. Grade 3 adverse events were defined as fever > 39.0°C; erythema and swelling with a diameter of >100 mm; injection-site pain, headache, fatigue, myalgia, arthralgia, chills, GI symptoms that prevented normal everyday activities. Error bars depict 95% CIs, which were based on the Clopper–Pearson CI method. Co-Ad group and control group received RZV dose 1 at day 1 (visit 1) and RZV dose 2 at day 61 (visit 3). Co-Ad group received the adjuvanted RSVPreF3 at day 1 (visit 1) and control group received the adjuvanted RSVPreF3 at day 31 (visit 2). Abbreviations: adjuvanted RSVPreF3, adjuvanted respiratory syncytial virus prefusion F protein-based vaccine; CI, confidence interval; Co-Ad group, co-administration of RZV dose 1 with adjuvanted RSVPreF3; Control group, sequential administration of RZV dose 1 and adjuvanted RSVPreF3; GI, gastrointestinal; RZV, adjuvanted recombinant herpes zoster subunit vaccine.

Overall, 80.4% (Gr3: 11.5%) of participants in the Co-Ad group and 79.2% (Gr3: 14.0%) of participants in the Control group reported ≥1 solicited systemic AE within 7 days post-any vaccination. Solicited systemic AEs were reported for 73.6% (Gr3: 9.3%) of participants within 7 days after co-administration of the 2 vaccines (Co-Ad group) and for 60.4% (Gr3: 6.2%) and 49.4% (Gr3: 3.7%) of participants within 7 days after separate administration of adjuvanted RSVPreF3 and RZV (Control group), respectively. Myalgia, fatigue, and headache were the most frequent solicited systemic AEs (Figure 6*B*; Supplementary Table 4). The median duration of each solicited systemic AE was ≤2 days.

Overall, a higher frequency of solicited AEs was observed in younger age groups (50–59 and 60–69 YOA) in both the Co-Ad and the control groups (Supplementary Tables 5 and 6).

The sensitivity analysis, conducted on the subsets of participants who completed all eDiary entries for solicited AEs for D1–3, D3–6, and D1–7, showed minor numerical differences for solicited AEs when compared with results from the end-of-study analysis.

In the ES, 23.4% (Gr3: 0.8%) of participants in the Co-Ad group and 30.2% (Gr3: 1.1%) of participants in the control group reported ≥1 unsolicited AE within 30 days post-any dose (Table 2). Thirteen (4.9%) participants in the Co-Ad group and 6 (2.3%) participants in the control group reported SAEs, none being fatal or related to vaccination (Table 2). Unsolicited AEs and SAEs stratified by age group are shown in Supplementary Table 7. One (0.4%) participant in the Co-Ad group, aged 60–69 years, with a history of Bell's palsy reported exacerbation of Bell's palsy 32 days post-co-administration of the 2 vaccines; this non-serious pIMD was considered related to vaccination by the investigator and resolved at study end. Two (0.8%) participants in the control group, both aged 50–59 years, reported gout; these pIMDs were not considered related to vaccination. No cases of Guillain–Barré syndrome, acute disseminated encephalomyelitis, or other neurological demyelinating events were reported.

**Table 2.** Summary of AEs (Exposed Set)

AEs	Co-Ad Group N = 265	Control Group N = 265
Unsolicited AEs within 30-d post-any vaccination, n (%)		
Any unsolicited AEs	62 (23.4)	80 (30.2)
Grade 3 unsolicited AEs	2 (0.8)	3 (1.1)
Related unsolicited AEs	12 (4.5)	12 (4.5)
Related grade 3 unsolicited AEs	0 (0.0)	0 (0.0)
SAEs and pIMDs up to study end, n (%)		
Any SAEs	13 (4.9)	6 (2.3)
Related SAEs	0 (0.0)	0 (0.0)
Any pIMDs	1 (0.4)	2 (0.8)
Related pIMDs	1 (0.4)	0 (0.0)

Abbreviations: adjuvanted RSVPreF3, adjuvanted respiratory syncytial virus prefusion F protein-based vaccine; AE, adverse event; Co-Ad group, co-administration of RZV dose 1 with adjuvanted RSVPreF3; Control group, sequential administration of RZV dose 1 and adjuvanted RSVPreF3; n (%), number (percentage) of participants in each group reporting the AE; N, total number of participants; pIMD, potential immune-mediated disease; RZV, adjuvanted recombinant herpes zoster subunit vaccine; SAE, serious AE.

## DISCUSSION

In this study, we showed that co-administration of adjuvanted RSVPreF3 and RZV was non-inferior to their sequential administration in terms of RSV-A/B neutralizing titers as well as VZV anti-gE antibody concentrations. Furthermore, we showed that co-administration was well tolerated, with clinically acceptable safety and reactogenicity profiles.

We conducted this study to assess the immune response, reactogenicity, and safety following co-administration of 2 AS01-adjuvanted vaccines. Previous phase III studies have assessed the immunogenicity and safety of adjuvanted RSVPreF3 when co-administered with standard-dose [25], high-dose [24], and MF59C.1-adjuvanted [26] seasonal influenza vaccines. The findings of these studies supported co-administration of RSV and influenza vaccines, which has now been incorporated in various national immunization programs [13, 28–30]. Other phase III, open-label, randomized trials have also shown that RZV co-administration with routine adult vaccines, like the quadrivalent seasonal inactivated influenza vaccine, 23-valent pneumococcal polysaccharide vaccine, reduced-antigen-content diphtheria-tetanus-acellular pertussis vaccine, 13-valent pneumococcal conjugate vaccine, and COVID-19 mRNA-based vaccine did not interfere with the immune response to either vaccine [31–35].

Co-administration of adjuvanted RSVPreF3 and RZV was well tolerated with an acceptable safety profile. Although there was a higher reporting frequency of overall solicited AEs within 7 days after co-administration of adjuvanted RSVPreF3 and first RZV dose (Co-Ad) versus separate administration of the 2 vaccines (Control), co-administration was not associated with a clinically relevant increased risk of solicited AEs. Additionally, the duration of solicited AEs was short and comparable between the 2 groups. The reporting frequency for any individual Gr3 solicited AE was acceptable, and no participant withdrew from the study due to Gr3 solicited AEs. The most frequently reported solicited AEs in both study groups were injection-site pain, myalgia, fatigue, and headache. Previous studies assessing the safety of co-administration of adjuvanted RSVPreF3 and influenza vaccines have also found injection-site pain, fatigue, headache, and myalgia as frequently reported solicited AEs [24–26]. The observed percentage of participants who experienced an unsolicited AE was slightly higher in the Control group (30.2%) versus Co-Ad group (23.4%), presumably due to the control group having one additional study intervention visit, thus providing another opportunity to capture AEs as well as a longer follow-up period. No deaths, related SAEs, cases of Guillain–Barré syndrome, or acute disseminated encephalomyelitis were reported.

To our knowledge, this is the first study assessing co-administration of 2 AS01-adjuvanted vaccines. The strength of this study includes the large sample size which allowed the assessment of safety endpoints and the participants' age range (≥50 YOA) which allowed investigators to detect the vaccines’ effects in a broader population. The sensitivity analysis found minor differences in the number of solicited AEs, with no impact on the safety conclusions.

A limitation of our study is the open-label design, which could have led to reporting bias of AEs. Secondly, the study did not investigate a schedule in which adjuvanted RSVPreF3 is administered prior to RZV. Also, our study was conducted exclusively in the North American population (the United States, Canada) and did not include immunocompromised individuals who are at risk of severe RSV disease and HZ. This study was not powered for age-stratified subgroup analyses, and thus, these results should be interpreted with caution. Compliance in completing the eDiaries was as low as 61%, as observed at visit 2 for the Control group. Lower compliance for the control group at visit 2 stemmed from delayed or occasional non-activation of eDiaries. Lack of timely reminders and unclear instructions provided to site staff contributed to delayed/non-activation of eDiaries.

In conclusion, this study has demonstrated that co-administration of adjuvanted RSVPreF3 and RZV induces an immune response that was non-inferior to sequential administration of these vaccines in adults ≥ 50 YOA, with clinically acceptable safety and reactogenicity profiles. Co-administration could improve vaccine coverage and reduce the number of vaccination visits. Our results support expanded recommendations.

## Supplementary Material

ciag208_Supplementary_Data

## Supplementary Data

Supplementary materials are available at *Clinical Infectious Diseases* online. Consisting of data provided by the authors to benefit the reader, the posted materials are not copyedited and are the sole responsibility of the authors, so questions or comments should be addressed to the corresponding author.
