# Supplementary material for: Immunogenicity and Safety of the Adjuvanted Respiratory Syncytial Virus Prefusion F Protein-Based Vaccine When Co-administered With the Adjuvanted Recombinant Herpes Zoster Subunit Vaccine in Adults ≥ 50 Years of Age
Source: Clin Infect Dis. 2026 Apr 29;83(3):e578–88. doi: 10.1093/cid/ciag208 (PMC13644881; doi:10.1093/cid/ciag208)
Supplement: Supplementary file 1 [file ciag208_supplementary_data.docx]

# **Immunogenicity and safety of the adjuvanted respiratory syncytial virus prefusion F protein-based vaccine when co-administered with the adjuvanted recombinant herpes zoster subunit vaccine in adults ≥50 years of age**

Supplementary material

[Supplementary methods	2](#_Toc222394549)

[1. Vaccine composition	2](#_Toc222394550)

[2. Inclusion and exclusion criteria	2](#_Toc222394551)

[3. Enrollment rules	6](#_Toc222394552)

[4. Clinical laboratory assays	7](#_Toc222394553)

[5. Clinical laboratory assay cut-offs	8](#_Toc222394554)

[6. Supplementary references	8](#_Toc222394555)

[Supplementary Table 1: Baseline characteristics of the participants (per-protocol set)	9](#_Toc222394556)

[Supplementary Table 2: Adjusted RSV-A/B neutralizing titer GMTs and anti-gE GMC ratios at 1M post-vaccination, by age group (per-protocol set)	10](#_Toc222394557)

[Supplementary Table 3: Compliance in completing the eDiary for solicited events (exposed set)	11](#_Toc222394558)

[Supplementary Table 4: Solicited administration-site and systemic AEs (exposed set)	12](#_Toc222394559)

[Supplementary Table 5: Proportion of participants with solicited administration-site AEs, by age group (exposed set)	15](#_Toc222394560)

[Supplementary Table 6: Proportion of participants with solicited systemic AEs, by age group (exposed set)	18](#_Toc222394561)

[Supplementary Table 7: Proportion of participants with unsolicited AEs and SAEs, by age group (exposed set)	22](#_Toc222394562)

# Supplementary methods

### 1. Vaccine composition

One dose of adjuvanted respiratory syncytial virus (RSV) prefusion F protein-based vaccine (adjuvanted RSVPreF3; 0.5 mL) contains 120 μg RSVPreF3 antigen and AS01_E_ adjuvant (25 μg MPL [3-*O*-desacyl-4′-monophosphoryl lipid A], 25 μg QS-21 [*Quillaja saponaria* Molina, fraction 21; licensed by GSK from Antigenics LLC, a wholly owned subsidiary of Agenus Inc., a Delaware, USA corporation], and liposomes) [1]. One dose of adjuvanted recombinant herpes zoster subunit vaccine (RZV; 0.5 mL) contains 50 μg of varicella-zoster virus glycoprotein E (gE) antigen and AS01_B_ adjuvant (50 μg MPL, 50 μg QS-21, and liposomes) [2].

### 2. Inclusion and exclusion criteria

**2.1 Inclusion criteria**

- A male or female participant ≥50 years of age at the time of the first study intervention administration.
- Female participants of non-childbearing potential (defined as hysterectomy, bilateral oophorectomy, bilateral salpingectomy, or post-menopause) could be enrolled in the study.
- Female participants of childbearing potential were eligible for enrollment in the study, if the participant:
  - had practiced adequate contraception from 1 month prior to study intervention administration.
  - had a negative pregnancy test on the day of and prior to study intervention administration.
  - had agreed to continue effective contraception until the end of the study.
- Participants who, in the opinion of the investigator, could and would comply with the requirements of the protocol (e.g., completion of the electronic diary [eDiary], return for follow-up visits, ability to access and utilize a phone or other electronic communications).

Note: In case of physical incapacity that would preclude the self-completion of the eDiary, either site staff could assist the participant (for activities performed during site visits) or the participant could assign a caregiver (i.e., a person who had a continuous caring role for a participant or had substantial periods of contact with a participant and/or was engaged in his/her daily health care [e.g., a relative of the participant including family members or friends]) to assist him/her with this activity (for activities performed at home). However, at no time would the site staff or caregiver evaluate the participant’s health status while answering eDiaries or make decisions on behalf of the participant.

- Written or witnessed informed consent obtained from the participant prior to any study-specific procedure being performed.
- Participants living in the general community or in an assisted-living facility that provided minimal assistance, such that the participant was primarily responsible for self-care and activities of daily living.
- Participants who were medically stable in the opinion of the investigator at the time of first study intervention administration. Participants with chronic stable medical conditions with or without specific treatment, such as diabetes mellitus, hypertension, or cardiac disease, were allowed to participate in this study if considered by the investigator as medically stable.

**2.2 Exclusion criteria**

*2.2.1 Medical conditions*

- Pregnant or lactating female.
- Female planning to become pregnant or planning to discontinue contraceptive precautions.
- Any confirmed or suspected autoimmune disorders, immunosuppressive or immunodeficient condition resulting from disease (e.g., current malignancy, human immunodeficiency virus), or immunosuppressive/cytotoxic therapy (e.g., medication used during cancer chemotherapy, organ transplantation, or to treat autoimmune disorders), based on medical history and physical examination (no laboratory testing required).
- History of any reaction or hypersensitivity (e.g., anaphylaxis) likely to be exacerbated by any component of the study interventions, in particular any history of severe allergic reaction to any vaccine component.
- History of Guillain-Barré syndrome.
- Any history of dementia or any medical condition that moderately or severely impaired cognition.

Note: If deemed necessary for clinical evaluation, the investigator could use tools such as Mini-Mental State Examination, Mini-Cog, or Montreal Cognitive Assessment to determine cognition levels of the participant.

- Recurrent or uncontrolled neurological disorders or seizures. Participants with medically controlled chronic neurological diseases could be enrolled in the study as per investigator assessment, provided that their condition would allow them to comply with the requirements of the protocol (e.g., completion of the eDiary, attending regular phone calls/study site visits).
- Significant underlying illness that in the opinion of the investigator would be expected to prevent completion of the study (e.g., life-threatening disease likely to limit survival up to study end).
- Any medical condition that in the judgment of the investigator would make intramuscular injection unsafe.
- Clinically suspected or polymerase chain reaction-confirmed ongoing episode of herpes zoster.

*2.2.2 Prior and concomitant therapy*

- Previous vaccination with licensed or investigational RSV vaccine.
- History of previous vaccination with any licensed or investigational adjuvanted recombinant herpes zoster vaccine before the study start or planned receipt through study participation.
- History of previous vaccination with any licensed or investigational live herpes zoster vaccine in the last 2 years from enrollment, or planned receipt through study participation.
- Use of any investigational or non-registered product (drug, vaccine, or medical device) other than the study interventions during the period beginning 30 days before the first dose of study interventions, or their planned use during the study period.
- Planned or actual administration of a vaccine not foreseen by the study protocol in the period starting 30 days before the first study intervention administration and ending 30 days after the last study intervention administration.
  - In the case of COVID-19 and inactivated/subunit/split influenza vaccines, this time window could be decreased to 14 days before and after each study intervention administration provided COVID-19 vaccine use was in line with local governmental recommendations.

Note: In case an emergency mass vaccination for an unforeseen public health threat (e.g., a pandemic) was recommended and/or organized by the public health authorities outside the routine immunization program, the time period described above could be reduced if necessary for that vaccine, provided it was used according to the local governmental recommendations and that the Sponsor was notified accordingly.

- Planned or actual administration of adjuvanted quadrivalent influenza vaccine not foreseen by the study protocol in the period starting 30 days before the first study intervention administration and ending 30 days after the last study intervention administration.
- Administration of long-acting immune-modifying drugs during the period starting 180 days before the administration of first dose of study interventions or planned administration at any time during the study period (e.g., infliximab).
- Administration of immunoglobulins and/or any blood products or plasma derivatives during the period starting 90 days before the administration of first dose of study interventions or planned administration during the study period.
- Chronic administration (defined as more than 14 consecutive days in total) of immunosuppressants or other immune-modifying drugs during the period starting 90 days prior to the first study intervention dose or planned administration during the study period.
  - For corticosteroids, this meant prednisone ≥20 mg/day, or equivalent.
  - Inhaled, topical, or intra-articular steroids were allowed.

*2.2.3 Prior/concurrent clinical study experience*

- Concurrently participating in another clinical study, at any time during the study period, in which the participant had been or would be exposed to an investigational or a non-investigational vaccine/product (drug or invasive medical device).

Note: European Economic Community (EEC) directive 93/42/EEC defines an invasive medical device as “*A device which, in whole or in part, penetrates inside the body, either through a body orifice or through the surface of the body”*.

*2.2.4 Other exclusions*

- History of chronic alcohol consumption and/or drug abuse as deemed by the investigator to render the potential participant unable/unlikely to provide accurate safety reports or comply with study procedures.
- Bedridden participants.
- Planned to move during the study conduct that prohibited participation until study end.
- Participation of any study personnel or their immediate dependents, family, or household members.

### 3. Enrollment rules

Overall, participants were enrolled in three age categories reflecting an approximate age distribution and disease burden in the general population with a balance between males and females. We enrolled approximately 30% of participants 50–59 years of age, approximately 30% of participants 60–69 years of age, and approximately 25% of participants ≥70 years of age. The remaining 15% were distributed freely across the three age categories. The age categories and percentage of participants in each age category were guided by the feasibility assessment. We also enrolled approximately 40% of participants from each sex; the remaining 20% of participants were distributed freely between both sexes.

### 4. Clinical laboratory assays

**4.1. Neutralization assay**

This functional assay measures the ability of serum to neutralize RSV entry and replicate in a host cell line. Virus neutralization is performed by incubating a fixed amount of RSV-A or RSV-B strain with serial dilutions of the test serum. The serum-virus mixture is then transferred onto a Vero cell culture and incubated for 2 days to allow infection of the Vero cells with the non-neutralized virus and the formation of plaques in the cell layer. Following a fixation step, RSV-infected cells are detected using a primary antibody directed against RSV and a secondary antibody conjugated to horse-radish peroxidase (HRP), to allow the visualization of plaques after coloration with a peroxidase substrate. Viral plaques are counted using an automated microscope coupled to an image analyzer. For each serum dilution, a ratio, expressed as a percentage, is calculated between the number of plaques in the test versus control wells. The serum neutralizing titer, expressed in estimated dilution 60 (ED_60_), corresponds to the inverse of the interpolated serum dilution that yields a 60% reduction in the number of plaques compared to the virus control wells [3, 4].

**4.2. Enzyme-linked immunosorbent assay (ELISA)**

The two-step ELISA, which is based on the antibody and antigen interaction, allows the detection and the quantification of specific IgG antibodies directed against varicella-zoster virus glycoprotein E (gE) in tested serum samples. Diluted serum samples are added onto a microplate pre-coated with gE. After a wash, goat antibodies directed against human IgG antibodies and conjugated to HRP are added. These antibodies bind to anti-gE IgG if present. After another wash, the addition of a chromogen-substrate solution specific for HRP provides means of detecting the anti-gE specific for the pre-coated antigen. The HRP catalyzes an enzymatic reaction which is stopped by the addition of sulfuric acid, resulting in color change from blue to yellow. The anti-gE antibody concentration present in the serum sample is estimated from optical density and is expressed in mIU/mL.

### 5. Clinical laboratory assay cut-offs

| **Assay** | **LLOQ value (unit)** | **ULOQ value (unit)** |
| --- | --- | --- |
| RSV-A neutralizing titers | 18 ED_60_ | 123,535 ED_60_ |
| RSV-B neutralizing titers | 30 ED_60_ | 138,336 ED_60_ |
| VZV anti-gE antibody concentrations | 97 mIU/mL | - |

**Abbreviations:** LLOQ, lower limit of quantification; ULOQ, upper limit of quantification; RSV, respiratory syncytial virus; ED_60_, estimated dilution 60; IU, International Units; VZV, varicella-zoster virus; gE, glycoprotein E.

### 6. Supplementary references

1.	European Medicines Agency. Arexvy: EPAR - Product Information. Available at: <https://www.ema.europa.eu/en/documents/product-information/arexvy-epar-product-information_en.pdf>. Accessed 14 Apr 2025.

2.	European Medicines Agency. Shingrix: EPAR - Product Information. Available at: <https://www.ema.europa.eu/en/documents/product-information/shingrix-epar-product-information_en.pdf>. Accessed 14 Apr 2025.

3.	Barbas CF, Crowe JE, Cababa D, et al. Human monoclonal Fab fragments derived from a combinatorial library bind to respiratory syncytial virus F glycoprotein and neutralize infectivity. Proc Natl Acad Sci USA **1992**; 89(21):10164-8.

4.	Bates JT, Keefer CJ, Slaughter JC, et al. Escape from neutralization by the respiratory syncytial virus-specific neutralizing monoclonal antibody palivizumab is driven by changes in on-rate of binding to the fusion protein. Virology **2014**; 454-455: 139-44.

### Supplementary Table 1: Baseline characteristics of the participants (per-protocol set)

| **Characteristic** | **Per-protocol set for RSV analysis** | | **Per-protocol set for HZ analysis** | |
| --- | --- | --- | --- | --- |
|  | **Co-Ad group**  **N=240** | **Control group**  **N=219** | **Co-Ad group**  **N=198** | **Control group**  **N=170** |
| **Age at first dose, mean (SD), years** | 63.9 (8.6) | 64.1 (9.0) | 64.2 (8.5) | 64.3 (8.7) |
| **Age at first dose, median (min–max), years** | 63.0 (50–87) | 63.0 (50–97) | 64.0 (50–87) | 64.0 (50–97) |
| **Age group, n (%)** |  |  |  |  |
| 50–59 years | 85 (35.4) | 79 (36.1) | 67 (33.8) | 59 (34.7) |
| 60–69 years | 84 (35.0) | 72 (32.9) | 69 (34.8) | 58 (34.1) |
| ≥70 years | 71 (29.6) | 68 (31.1) | 62 (31.3) | 53 (31.2) |
| ≥75 years | 30 (12.5) | 23 (10.5) | 27 (13.6) | 16 (9.4) |
| **Sex, n (%)** |  |  |  |  |
| Male | 107 (44.6) | 109 (49.8) | 84 (42.4) | 83 (48.8) |
| Female | 133 (55.4) | 110 (50.2) | 114 (57.6) | 87 (51.2) |
| **Country, n (%)** |  |  |  |  |
| Canada | 158 (65.8) | 150 (68.5) | 127 (64.1) | 109 (64.1) |
| US | 82 (34.2) | 69 (31.5) | 71 (35.9) | 61 (35.9) |
| **Race, n (%)** |  |  |  |  |
| Black or African American | 37 (15.4) | 33 (15.1) | 29 (14.6) | 25 (14.7) |
| American Indian or Alaska Native | 3 (1.3) | 1 (0.5) | 3 (1.5) | 1 (0.6) |
| Asian | 32 (13.3) | 29 (13.2) | 8 (4.0) | 7 (4.1) |
| Native Hawaiian or Other Pacific Islanders | 1 (0.4) | 0 (0.0) | 0 (0.0) | 0 (0.0) |
| White | 163 (67.9) | 150 (68.5) | 154 (77.8) | 133 (78.2) |
| Multiple | 1 (0.4) | 2 (0.9) | 1 (0.5) | 2 (1.2) |
| Other | 1 (0.4) | 3 (1.4) | 1 (0.5) | 2 (1.2) |
| Missing | 2 (0.8) | 1 (0.5) | 2 (1.0) | 0 (0.0) |
| **Ethnicity, n (%)** |  |  |  |  |
| Hispanic or Latino | 25 (10.4) | 25 (11.4) | 22 (11.1) | 21 (12.4) |

**Abbreviations:** RSV, respiratory syncytial virus; HZ, herpes zoster; Co-Ad group, co-administration of RZV dose 1 with adjuvanted RSVPreF3; RZV, adjuvanted recombinant herpes zoster subunit vaccine; adjuvanted RSVPreF3, adjuvanted respiratory syncytial virus prefusion F protein-based vaccine; N, total number of participants; Control group, sequential administration of RZV dose 1 and adjuvanted RSVPreF3; SD, standard deviation; n (%), number (percentage) of participants in each group with the specified characteristic; US, United States.

### Supplementary Table 2: Adjusted RSV-A/B neutralizing titer GMTs and anti-gE GMC ratios at 1M post-vaccination, by age group (per-protocol set)

|  |  | **Adjusted GMT/GMC ratio (95% CI)** | | |
| --- | --- | --- | --- | --- |
|  |  | **50–59 YOA** | **60–69 YOA** | **≥70 YOA** |
| 1M post-adjuvanted RSVPreF3 | RSV-A | 1.4 (1.1–1.8) | 1.1 (0.8–1.4) | 1.0 (0.7–1.4) |
|  | RSV-B | 1.2 (1.0–1.5) | 0.8 (0.6–1.0) | 0.9 (0.6–1.3) |
| 1M post-RZV dose 2 | VZV anti-gE | 1.3 (1.1–1.7) | 1.3 (1.0–1.6) | 1.1 (0.9–1.5) |

**Abbreviations:** RSV-A/B, respiratory syncytial virus subtype A/B; GMT, geometric mean titer; gE, glycoprotein E; GMC, geometric mean concentration; M, month; CI, confidence interval; YOA, years of age; adjuvanted RSVPreF3, adjuvanted respiratory syncytial virus prefusion F protein-based vaccine; RZV, adjuvanted recombinant herpes zoster subunit vaccine; VZV, varicella-zoster virus.

### Supplementary Table 3: Compliance in completing the eDiary for solicited events (exposed set)

|  |  | **Co-Ad group** | | | **Control group** | | |
| --- | --- | --- | --- | --- | --- | --- | --- |
|  |  | **N** | **n** | **%** | **N** | **n** | **%** |
| Visit 1 | Day 1 | 265 | 237 | 89.4 | 265 | 241 | 90.9 |
|  | Day 2 | 265 | 245 | 92.5 | 265 | 248 | 93.6 |
|  | Day 3 | 265 | 240 | 90.6 | 265 | 241 | 90.9 |
|  | Day 4 | 265 | 231 | 87.2 | 265 | 244 | 92.1 |
|  | Day 5 | 265 | 236 | 89.1 | 265 | 244 | 92.1 |
|  | Day 6 | 265 | 231 | 87.2 | 265 | 232 | 87.5 |
|  | Day 7 | 265 | 231 | 87.2 | 265 | 235 | 88.7 |
| Visit 2 | Day 1 | NA | NA | NA | 261 | 158 | 60.5 |
|  | Day 2 | NA | NA | NA | 261 | 207 | 79.3 |
|  | Day 3 | NA | NA | NA | 261 | 212 | 81.2 |
|  | Day 4 | NA | NA | NA | 261 | 223 | 85.4 |
|  | Day 5 | NA | NA | NA | 261 | 209 | 80.1 |
|  | Day 6 | NA | NA | NA | 261 | 218 | 83.5 |
|  | Day 7 | NA | NA | NA | 261 | 212 | 81.2 |
| Visit 3 | Day 1 | 259 | 189 | 73.0 | 258 | 200 | 77.5 |
|  | Day 2 | 259 | 210 | 81.1 | 258 | 219 | 84.9 |
|  | Day 3 | 259 | 210 | 81.1 | 258 | 223 | 86.4 |
|  | Day 4 | 259 | 208 | 80.3 | 258 | 219 | 84.9 |
|  | Day 5 | 259 | 211 | 81.5 | 258 | 220 | 85.3 |
|  | Day 6 | 259 | 198 | 76.4 | 258 | 205 | 79.5 |
|  | Day 7 | 259 | 200 | 77.2 | 258 | 217 | 84.1 |

**Abbreviations:** eDiary, electronic diary; Co-Ad group, co-administration of RZV dose 1 with adjuvanted RSVPreF3; RZV, adjuvanted recombinant herpes zoster subunit vaccine; adjuvanted RSVPreF3, adjuvanted respiratory syncytial virus prefusion F protein-based vaccine; Control group, sequential administration of RZV dose 1 and adjuvanted RSVPreF3; N, total number of participants; n (%), number (percentage) of participants in each group; NA, not applicable.

### Supplementary Table 4: Solicited administration-site and systemic AEs (exposed set)

| AE | Intervention/timepoint | Intensity | Co-Ad group | | | Control group | | |
| --- | --- | --- | --- | --- | --- | --- | --- | --- |
|  |  |  | **N** | **n** | **% (95% CI^a^)** | **N** | **n** | **% (95% CI^a^)** |
| Administration-site | | | | | | | | |
| Injection-site pain | RZV dose 1^b^ | Any | 253 | 171 | 67.6 (61.4–73.3) | 251 | 150 | 59.8 (53.4–65.9) |
|  |  | Grade 3 |  | 6 | 2.4 (0.9–5.1) |  | 2 | 0.8 (0.1–2.8) |
|  | Adjuvanted RSVPreF3^c^ | Any | 252 | 159 | 63.1 (56.8–69.1) | 236 | 117 | 49.6 (43.0–56.1) |
|  |  | Grade 3 |  | 5 | 2.0 (0.6–4.6) |  | 3 | 1.3 (0.3–3.7) |
|  | RZV dose 2^d^ | Any | 225 | 129 | 57.3 (50.6–63.9) | 235 | 149 | 63.4 (56.9–69.6) |
|  |  | Grade 3 |  | 1 | 0.4 (0.0–2.5) |  | 3 | 1.3 (0.3–3.7) |
|  | Per participant | Any | 255 | 199 | 78.0 (72.5–83.0) | 255 | 191 | 74.9 (69.1–80.1) |
|  |  | Grade 3 |  | 8 | 3.1 (1.4–6.1) |  | 6 | 2.4 (0.9–5.1) |
| Erythema | RZV dose 1^b^ | Any | 253 | 37 | 14.6 (10.5–19.6) | 251 | 33 | 13.1 (9.2–18.0) |
|  |  | Grade 3 |  | 1 | 0.4 (0.0–2.2) |  | 2 | 0.8 (0.1–2.8) |
|  | Adjuvanted RSVPreF3^c^ | Any | 252 | 20 | 7.9 (4.9–12.0) | 236 | 18 | 7.6 (4.6–11.8) |
|  |  | Grade 3 |  | 0 | 0.0 (0.0–1.5) |  | 0 | 0.0 (0.0–1.6) |
|  | RZV dose 2^d^ | Any | 225 | 36 | 16.0 (11.5–21.5) | 235 | 28 | 11.9 (8.1–16.8) |
|  |  | Grade 3 |  | 1 | 0.4 (0.0–2.5) |  | 0 | 0.0 (0.0–1.6) |
|  | Per participant | Any | 255 | 57 | 22.4 (17.4–28.0) | 255 | 53 | 20.8 (16.0–26.3) |
|  |  | Grade 3 |  | 2 | 0.8 (0.1–2.8) |  | 2 | 0.8 (0.1–2.8) |
| Swelling | RZV dose 1^b^ | Any | 253 | 22 | 8.7 (5.5–12.9) | 251 | 22 | 8.8 (5.6–13.0) |
|  |  | Grade 3 |  | 1 | 0.4 (0.0–2.2) |  | 1 | 0.4 (0.0–2.2) |
|  | Adjuvanted RSVPreF3^c^ | Any | 252 | 15 | 6.0 (3.4–9.6) | 236 | 13 | 5.5 (3.0–9.2) |
|  |  | Grade 3 |  | 0 | 0.0 (0.0–1.5) |  | 0 | 0.0 (0.0–1.6) |
|  | RZV dose 2^d^ | Any | 225 | 20 | 8.9 (5.5–13.4) | 235 | 20 | 8.5 (5.3–12.8) |
|  |  | Grade 3 |  | 0 | 0.0 (0.0–1.6) |  | 1 | 0.4 (0.0–2.3) |
|  | Per participant | Any | 255 | 37 | 14.5 (10.4–19.4) | 255 | 36 | 14.1 (10.1–19.0) |
|  |  | Grade 3 |  | 1 | 0.4 (0.0–2.2) |  | 2 | 0.8 (0.1–2.8) |
| Systemic | | | | | | | | |
| Fever | Visit 1 | Any | 258 | 23 | 8.9 (5.7–13.1) | 260 | 16 | 6.2 (3.6–9.8) |
|  |  | Grade 3 |  | 8 | 3.1 (1.3–6.0) |  | 5 | 1.9 (0.6–4.4) |
|  | Visit 2 | Any |  |  |  | 245 | 8 | 3.3 (1.4–6.3) |
|  |  | Grade 3 |  |  |  |  | 2 | 0.8 (0.1–2.9) |
|  | Visit 3 | Any | 230 | 7 | 3.0 (1.2–6.2) | 243 | 18 | 7.4 (4.4–11.5) |
|  |  | Grade 3 |  | 1 | 0.4 (0.0–2.4) |  | 2 | 0.8 (0.1–2.9) |
|  | Per participant | Any | 260 | 29 | 11.2 (7.6–15.6) | 264 | 39 | 14.8 (10.7–19.6) |
|  |  | Grade 3 |  | 9 | 3.5 (1.6–6.5) |  | 9 | 3.4 (1.6–6.4) |
| Headache | Visit 1 | Any | 258 | 96 | 37.2 (31.3–43.4) | 260 | 78 | 30.0 (24.5–36.0) |
|  |  | Grade 3 |  | 5 | 1.9 (0.6–4.5) |  | 4 | 1.5 (0.4–3.9) |
|  | Visit 2 | Any |  |  |  | 245 | 56 | 22.9 (17.8–28.6) |
|  |  | Grade 3 |  |  |  |  | 1 | 0.4 (0.0–2.3) |
|  | Visit 3 | Any | 230 | 71 | 30.9 (25.0–37.3) | 243 | 81 | 33.3 (27.4–39.6) |
|  |  | Grade 3 |  | 4 | 1.7 (0.5–4.4) |  | 3 | 1.2 (0.3–3.6) |
|  | Per participant | Any | 260 | 119 | 45.8 (39.6–52.0) | 264 | 138 | 52.3 (46.1–58.4) |
|  |  | Grade 3 |  | 7 | 2.7 (1.1–5.5) |  | 8 | 3.0 (1.3–5.9) |
| Fatigue | Visit 1 | Any | 258 | 126 | 48.8 (42.6–55.1) | 260 | 94 | 36.2 (30.3–42.3) |
|  |  | Grade 3 |  | 8 | 3.1 (1.3–6.0) |  | 3 | 1.2 (0.2–3.3) |
|  | Visit 2 | Any |  |  |  | 245 | 78 | 31.8 (26.1–38.1) |
|  |  | Grade 3 |  |  |  |  | 4 | 1.6 (0.4–4.1) |
|  | Visit 3 | Any | 230 | 91 | 39.6 (33.2–46.2) | 243 | 113 | 46.5 (40.1–53.0) |
|  |  | Grade 3 |  | 3 | 1.3 (0.3–3.8) |  | 9 | 3.7 (1.7–6.9) |
|  | Per participant | Any | 260 | 149 | 57.3 (51.0–63.4) | 264 | 156 | 59.1 (52.9–65.1) |
|  |  | Grade 3 |  | 10 | 3.8 (1.9–7.0) |  | 16 | 6.1 (3.5–9.7) |
| Myalgia | Visit 1 | Any | 258 | 134 | 51.9 (45.7–58.2) | 260 | 105 | 40.4 (34.4–46.6) |
|  |  | Grade 3 |  | 4 | 1.6 (0.4–3.9) |  | 3 | 1.2 (0.2–3.3) |
|  | Visit 2 | Any |  |  |  | 245 | 66 | 26.9 (21.5–33.0) |
|  |  | Grade 3 |  |  |  |  | 4 | 1.6 (0.4–4.1) |
|  | Visit 3 | Any | 230 | 85 | 37.0 (30.7–43.5) | 243 | 101 | 41.6 (35.3–48.0) |
|  |  | Grade 3 |  | 3 | 1.3 (0.3–3.8) |  | 4 | 1.6 (0.5–4.2) |
|  | Per participant | Any | 260 | 153 | 58.8 (52.6–64.9) | 264 | 154 | 58.3 (52.1–64.3) |
|  |  | Grade 3 |  | 6 | 2.3 (0.9–5.0) |  | 9 | 3.4 (1.6–6.4) |
| Arthralgia | Visit 1 | Any | 258 | 62 | 24.0 (18.9–29.7) | 260 | 42 | 16.2 (11.9–21.2) |
|  |  | Grade 3 |  | 2 | 0.8 (0.1–2.8) |  | 0 | 0.0 (0.0–1.4) |
|  | Visit 2 | Any |  |  |  | 245 | 31 | 12.7 (8.8–17.5) |
|  |  | Grade 3 |  |  |  |  | 4 | 1.6 (0.4–4.1) |
|  | Visit 3 | Any | 230 | 48 | 20.9 (15.8–26.7) | 243 | 51 | 21.0 (16.0–26.7) |
|  |  | Grade 3 |  | 4 | 1.7 (0.5–4.4) |  | 2 | 0.8 (0.1–2.9) |
|  | Per participant | Any | 260 | 85 | 32.7 (27.0–38.8) | 264 | 78 | 29.5 (24.1–35.4) |
|  |  | Grade 3 |  | 6 | 2.3 (0.9–5.0) |  | 6 | 2.3 (0.8–4.9) |
| Chills | Visit 1 | Any | 258 | 59 | 22.9 (17.9–28.5) | 260 | 33 | 12.7 (8.9–17.4) |
|  |  | Grade 3 |  | 4 | 1.6 (0.4–3.9) |  | 0 | 0.0 (0.0–1.4) |
|  | Visit 2 | Any |  |  |  | 245 | 32 | 13.1 (9.1–17.9) |
|  |  | Grade 3 |  |  |  |  | 3 | 1.2 (0.3–3.5) |
|  | Visit 3 | Any | 230 | 53 | 23.0 (17.8–29.0) | 243 | 63 | 25.9 (20.5–31.9) |
|  |  | Grade 3 |  | 3 | 1.3 (0.3–3.8) |  | 5 | 2.1 (0.7–4.7) |
|  | Per participant | Any | 260 | 85 | 32.7 (27.0–38.8) | 264 | 90 | 34.1 (28.4–40.2) |
|  |  | Grade 3 |  | 7 | 2.7 (1.1–5.5) |  | 8 | 3.0 (1.3–5.9) |
| GI symptoms | Visit 1 | Any | 258 | 41 | 15.9 (11.7–20.9) | 260 | 39 | 15.0 (10.9–19.9) |
|  |  | Grade 3 |  | 2 | 0.8 (0.1–2.8) |  | 4 | 1.5 (0.4–3.9) |
|  | Visit 2 | Any |  |  |  | 245 | 28 | 11.4 (7.7–16.1) |
|  |  | Grade 3 |  |  |  |  | 2 | 0.8 (0.1–2.9) |
|  | Visit 3 | Any | 230 | 21 | 9.1 (5.7–13.6) | 243 | 30 | 12.3 (8.5–17.2) |
|  |  | Grade 3 |  | 0 | 0.0 (0.0–1.6) |  | 0 | 0.0 (0.0–1.5) |
|  | Per participant | Any | 260 | 54 | 20.8 (16.0–26.2) | 264 | 67 | 25.4 (20.2–31.1) |
|  |  | Grade 3 |  | 2 | 0.8 (0.1–2.8) |  | 5 | 1.9 (0.6–4.4) |

**Notes:** Fever was defined as a temperature ≥38.0°C. Grade 3 AEs were defined as fever >39.0°C; erythema and swelling with a diameter of >100 mm; injection-site pain, headache, fatigue, myalgia, arthralgia, chills, GI symptoms that prevented normal everyday activities. ^a^95% CIs were based on the Clopper-Pearson CI method. ^b^Co-Ad group and Control group received RZV dose 1 at Day 1 (Visit 1). ^c^Co-Ad group received the adjuvanted RSVPreF3 at Day 1 (Visit 1) and Control group received the adjuvanted RSVPreF3 at Day 31 (Visit 2). ^d^Co-Ad group and Control group received RZV dose 2 at Day 61 (Visit 3).

**Abbreviations:** AE, adverse event; Co-Ad group, co-administration of RZV dose 1 with adjuvanted RSVPreF3; Control group, sequential administration of RZV dose 1 and adjuvanted RSVPreF3; N, number of participants with completed eDiary card; eDiary, electronic diary; n (%), number (percentage) of participants in each group with completed eDiary cards presenting at least one type of symptom; CI, confidence interval; RZV, adjuvanted recombinant herpes zoster subunit vaccine; adjuvanted RSVPreF3, adjuvanted respiratory syncytial virus prefusion F protein-based vaccine; GI, gastrointestinal.

### Supplementary Table 5: Proportion of participants with solicited administration-site AEs, by age group (exposed set)

| AE | Intervention/  timepoint | Intensity | Co-Ad group | | | Control group | | |
| --- | --- | --- | --- | --- | --- | --- | --- | --- |
|  |  |  | **N** | **n** | **% (95% CI^a^)** | **N** | **n** | **% (95% CI^a^)** |
| 50–59 YOA | | | | | | | | |
| Injection-site pain | RZV dose 1^b^ | Any | 90 | 70 | 77.8 (67.8–85.9) | 90 | 59 | 65.6 (54.8–75.3) |
|  |  | Grade 3 |  | 5 | 5.6 (1.8–12.5) |  | 1 | 1.1 (0.0–6.0) |
|  | Adjuvanted RSVPreF3^c^ | Any | 90 | 67 | 74.4 (64.2–83.1) | 85 | 47 | 55.3 (44.1–66.1) |
|  |  | Grade 3 |  | 3 | 3.3 (0.7–9.4) |  | 2 | 2.4 (0.3–8.2) |
|  | RZV dose 2^d^ | Any | 76 | 55 | 72.4 (60.9–82.0) | 79 | 57 | 72.2 (60.9–81.7) |
|  |  | Grade 3 |  | 1 | 1.3 (0.0–7.1) |  | 3 | 3.8 (0.8–10.7) |
|  | Per participant | Any | 90 | 76 | 84.4 (75.3–91.2) | 91 | 72 | 79.1 (69.3–86.9) |
|  |  | Grade 3 |  | 6 | 6.7 (2.5–13.9) |  | 4 | 4.4 (1.2–10.9) |
| Erythema | RZV dose 1^b^ | Any | 90 | 23 | 25.6 (16.9–35.8) | 90 | 21 | 23.3 (15.1–33.4) |
|  |  | Grade 3 |  | 0 | 0.0 (0.0–4.0) |  | 0 | 0.0 (0.0–4.0) |
|  | Adjuvanted RSVPreF3^c^ | Any | 90 | 14 | 15.6 (8.8–24.7) | 85 | 15 | 17.6 (10.2–27.4) |
|  |  | Grade 3 |  | 0 | 0.0 (0.0–4.0) |  | 0 | 0.0 (0.0–4.2) |
|  | RZV dose 2^d^ | Any | 76 | 19 | 25.0 (15.8–36.3) | 79 | 18 | 22.8 (14.1–33.6) |
|  |  | Grade 3 |  | 0 | 0.0 (0.0–4.7) |  | 0 | 0.0 (0.0–4.6) |
|  | Per participant | Any | 90 | 34 | 37.8 (27.8–48.6) | 91 | 32 | 35.2 (25.4–45.9) |
|  |  | Grade 3 |  | 0 | 0.0 (0.0–4.0) |  | 0 | 0.0 (0.0–4.0) |
| Swelling | RZV dose 1^b^ | Any | 90 | 19 | 21.1 (13.2–31.0) | 90 | 19 | 21.1 (13.2–31.0) |
|  |  | Grade 3 |  | 0 | 0.0 (0.0–4.0) |  | 0 | 0.0 (0.0–4.0) |
|  | Adjuvanted RSVPreF3^c^ | Any | 90 | 14 | 15.6 (8.8–24.7) | 85 | 13 | 15.3 (8.4–24.7) |
|  |  | Grade 3 |  | 0 | 0.0 (0.0–4.0) |  | 0 | 0.0 (0.0–4.2) |
|  | RZV dose 2^d^ | Any | 76 | 19 | 25.0 (15.8–36.3) | 79 | 13 | 16.5 (9.1–26.5) |
|  |  | Grade 3 |  | 0 | 0.0 (0.0–4.7) |  | 0 | 0.0 (0.0–4.6) |
|  | Per participant | Any | 90 | 32 | 35.6 (25.7–46.3) | 91 | 30 | 33.0 (23.5–43.6) |
|  |  | Grade 3 |  | 0 | 0.0 (0.0–4.0) |  | 0 | 0.0 (0.0–4.0) |
| 60–69 YOA | | | | | | | | |
| Injection-site pain | RZV dose 1^b^ | Any | 88 | 58 | 65.9 (55.0–75.7) | 88 | 57 | 64.8 (53.9–74.7) |
|  |  | Grade 3 |  | 0 | 0.0 (0.0–4.1) |  | 1 | 1.1 (0.0–6.2) |
|  | Adjuvanted RSVPreF3^c^ | Any | 88 | 57 | 64.8 (53.9–74.7) | 83 | 40 | 48.2 (37.1–59.4) |
|  |  | Grade 3 |  | 1 | 1.1 (0.0–6.2) |  | 1 | 1.2 (0.0–6.5) |
|  | RZV dose 2^d^ | Any | 79 | 46 | 58.2 (46.6–69.2) | 87 | 59 | 67.8 (56.9–77.4) |
|  |  | Grade 3 |  | 0 | 0.0 (0.0–4.6) |  | 0 | 0.0 (0.0–4.2) |
|  | Per participant | Any | 89 | 72 | 80.9 (71.2–88.5) | 89 | 69 | 77.5 (67.4–85.7) |
|  |  | Grade 3 |  | 1 | 1.1 (0.0–6.1) |  | 2 | 2.2 (0.3–7.9) |
| Erythema | RZV dose 1^b^ | Any | 88 | 19 | 21.6 (13.5–31.6) | 88 | 24 | 27.3 (18.3–37.8) |
|  |  | Grade 3 |  | 1 | 1.1 (0.0–6.2) |  | 1 | 1.1 (0.0–6.2) |
|  | Adjuvanted RSVPreF3^c^ | Any | 88 | 18 | 20.5 (12.6–30.4) | 83 | 8 | 9.6 (4.3–18.1) |
|  |  | Grade 3 |  | 0 | 0.0 (0.0–4.1) |  | 0 | 0.0 (0.0–4.3) |
|  | RZV dose 2^d^ | Any | 79 | 21 | 26.6 (17.3–37.7) | 87 | 16 | 18.4 (10.9–28.1) |
|  |  | Grade 3 |  | 1 | 1.3 (0.0–6.9) |  | 0 | 0.0 (0.0–4.2) |
|  | Per participant | Any | 89 | 33 | 37.1 (27.1–48.0) | 89 | 30 | 33.7 (24.0–44.5) |
|  |  | Grade 3 |  | 2 | 2.2 (0.3–7.9) |  | 1 | 1.1 (0.0–6.1) |
| Swelling | RZV dose 1^b^ | Any | 88 | 15 | 17.0 (9.9–26.6) | 88 | 19 | 21.6 (13.5–31.6) |
|  |  | Grade 3 |  | 1 | 1.1 (0.0–6.2) |  | 1 | 1.1 (0.0–6.2) |
|  | Adjuvanted RSVPreF3^c^ | Any | 88 | 13 | 14.8 (8.1–23.9) | 83 | 12 | 14.5 (7.7–23.9) |
|  |  | Grade 3 |  | 0 | 0.0 (0.0–4.1) |  | 0 | 0.0 (0.0–4.3) |
|  | RZV dose 2^d^ | Any | 79 | 17 | 21.5 (13.1–32.2) | 87 | 19 | 21.8 (13.7–32.0) |
|  |  | Grade 3 |  | 0 | 0.0 (0.0–4.6) |  | 0 | 0.0 (0.0–4.2) |
|  | Per participant | Any | 89 | 26 | 29.2 (20.1–39.8) | 89 | 29 | 32.6 (23.0–43.3) |
|  |  | Grade 3 |  | 1 | 1.1 (0.0–6.1) |  | 1 | 1.1 (0.0–6.1) |
| ≥70 YOA | | | | | | | | |
| Injection-site pain | RZV dose 1^b^ | Any | 75 | 43 | 57.3 (45.4–68.7) | 73 | 34 | 46.6 (34.8–58.6) |
|  |  | Grade 3 |  | 1 | 1.3 (0.0–7.2) |  | 0 | 0.0 (0.0–4.9) |
|  | Adjuvanted RSVPreF3^c^ | Any | 74 | 35 | 47.3 (35.6–59.3) | 68 | 30 | 44.1 (32.1–56.7) |
|  |  | Grade 3 |  | 1 | 1.4 (0.0–7.3) |  | 0 | 0.0 (0.0–5.3) |
|  | RZV dose 2^d^ | Any | 70 | 28 | 40.0 (28.5–52.4) | 69 | 33 | 47.8 (35.6–60.2) |
|  |  | Grade 3 |  | 0 | 0.0 (0.0–5.1) |  | 0 | 0.0 (0.0–5.2) |
|  | Per participant | Any | 76 | 51 | 67.1 (55.4–77.5) | 75 | 50 | 66.7 (54.8–77.1) |
|  |  | Grade 3 |  | 1 | 1.3 (0.0–7.1) |  | 0 | 0.0 (0.0–4.8) |
| Erythema | RZV dose 1^b^ | Any | 75 | 12 | 16.0 (8.6–26.3) | 73 | 21 | 28.8 (18.8–40.6) |
|  |  | Grade 3 |  | 0 | 0.0 (0.0–4.8) |  | 1 | 1.4 (0.0–7.4) |
|  | Adjuvanted RSVPreF3^c^ | Any | 74 | 7 | 9.5 (3.9–18.5) | 68 | 9 | 13.2 (6.2–23.6) |
|  |  | Grade 3 |  | 0 | 0.0 (0.0–4.9) |  | 0 | 0.0 (0.0–5.3) |
|  | RZV dose 2^d^ | Any | 70 | 9 | 12.9 (6.1–23.0) | 69 | 13 | 18.8 (10.4–30.1) |
|  |  | Grade 3 |  | 0 | 0.0 (0.0–5.1) |  | 0 | 0.0 (0.0–5.2) |
|  | Per participant | Any | 76 | 16 | 21.1 (12.5–31.9) | 75 | 25 | 33.3 (22.9–45.2) |
|  |  | Grade 3 |  | 0 | 0.0 (0.0–4.7) |  | 1 | 1.3 (0.0–7.2) |
| Swelling | RZV dose 1^b^ | Any | 75 | 14 | 18.7 (10.6–29.3) | 73 | 14 | 19.2 (10.9–30.1) |
|  |  | Grade 3 |  | 0 | 0.0 (0.0–4.8) |  | 0 | 0.0 (0.0–4.9) |
|  | Adjuvanted RSVPreF3^c^ | Any | 74 | 10 | 13.5 (6.7–23.5) | 68 | 6 | 8.8 (3.3–18.2) |
|  |  | Grade 3 |  | 0 | 0.0 (0.0–4.9) |  | 0 | 0.0 (0.0–5.3) |
|  | RZV dose 2^d^ | Any | 70 | 10 | 14.3 (7.1–24.7) | 69 | 12 | 17.4 (9.3–28.4) |
|  |  | Grade 3 |  | 0 | 0.0 (0.0–5.1) |  | 1 | 1.4 (0.0–7.8) |
|  | Per participant | Any | 76 | 20 | 26.3 (16.9–37.7) | 75 | 18 | 24.0 (14.9–35.3) |
|  |  | Grade 3 |  | 0 | 0.0 (0.0–4.7) |  | 1 | 1.3 (0.0–7.2) |

**Notes:** Grade 3 AEs were defined as erythema and swelling with a diameter of >100 mm and injection-site pain that prevented normal everyday activities. ^a^95% CIs were based on the Clopper-Pearson CI method. ^b^Co-Ad group and Control group received RZV dose 1 at Day 1 (Visit 1). ^c^Co-Ad group received the adjuvanted RSVPreF3 at Day 1 (Visit 1) and Control group received the adjuvanted RSVPreF3 at Day 31 (Visit 2). ^d^Co-Ad group and Control group received RZV dose 2 at Day 61 (Visit 3).

**Abbreviations:** AE, adverse event; Co-Ad group, co-administration of RZV dose 1 with adjuvanted RSVPreF3; Control group, sequential administration of RZV dose 1 and adjuvanted RSVPreF3; N, number of participants with completed eDiary card; eDiary, electronic diary; n (%), number (percentage) of participants in each group with completed eDiary cards presenting at least one type of symptom; CI, confidence interval; YOA, years of age; RZV, adjuvanted recombinant herpes zoster subunit vaccine; adjuvanted RSVPreF3, adjuvanted respiratory syncytial virus prefusion F protein-based vaccine.

### Supplementary Table 6: Proportion of participants with solicited systemic AEs, by age group (exposed set)

| AE | Intervention/  timepoint | Intensity | Co-Ad group | | | Control group | | |
| --- | --- | --- | --- | --- | --- | --- | --- | --- |
|  |  |  | **N** | **n** | **% (95% CI^a^)** | **N** | **n** | **% (95% CI^a^)** |
| 50–59 YOA | | | | | | | | |
| Fever | Visit 1 | Any | 92 | 8 | 8.7 (3.8–16.4) | 93 | 4 | 4.3 (1.2–10.6) |
|  |  | Grade 3 |  | 1 | 1.1 (0.0–5.9) |  | 1 | 1.1 (0.0–5.8) |
|  | Visit 2 | Any |  |  |  | 88 | 5 | 5.7 (1.9–12.8) |
|  |  | Grade 3 |  |  |  |  | 1 | 1.1 (0.0–6.2) |
|  | Visit 3 | Any | 78 | 5 | 6.4 (2.1–14.3) | 82 | 9 | 11.0 (5.1–19.8) |
|  |  | Grade 3 |  | 1 | 1.3 (0.0–6.9) |  | 1 | 1.2 (0.0–6.6) |
|  | Per participant | Any | 92 | 12 | 13.0 (6.9–21.7) | 94 | 16 | 17.0 (10.1–26.2) |
|  |  | Grade 3 |  | 2 | 2.2 (0.3–7.6) |  | 3 | 3.2 (0.7–9.0) |
| Headache | Visit 1 | Any | 92 | 43 | 46.7 (36.3–57.4) | 93 | 35 | 37.6 (27.8–48.3) |
|  |  | Grade 3 |  | 4 | 4.3 (1.2–10.8) |  | 2 | 2.2 (0.3–7.6) |
|  | Visit 2 | Any |  |  |  | 88 | 24 | 27.3 (18.3–37.8) |
|  |  | Grade 3 |  |  |  |  | 1 | 1.1 (0.0–6.2) |
|  | Visit 3 | Any | 78 | 34 | 43.6 (32.4–55.3) | 82 | 32 | 39.0 (28.4–50.4) |
|  |  | Grade 3 |  | 4 | 5.1 (1.4–12.6) |  | 2 | 2.4 (0.3–8.5) |
|  | Per participant | Any | 92 | 52 | 56.5 (45.8–66.8) | 94 | 57 | 60.6 (50.0–70.6) |
|  |  | Grade 3 |  | 6 | 6.5 (2.4–13.7) |  | 5 | 5.3 (1.7–12.0) |
| Fatigue | Visit 1 | Any | 92 | 52 | 56.5 (45.8–66.8) | 93 | 46 | 49.5 (38.9–60.0) |
|  |  | Grade 3 |  | 5 | 5.4 (1.8–12.2) |  | 1 | 1.1 (0.0–5.8) |
|  | Visit 2 | Any |  |  |  | 88 | 36 | 40.9 (30.5–51.9) |
|  |  | Grade 3 |  |  |  |  | 3 | 3.4 (0.7–9.6) |
|  | Visit 3 | Any | 78 | 40 | 51.3 (39.7–62.8) | 82 | 40 | 48.8 (37.6–60.1) |
|  |  | Grade 3 |  | 2 | 2.6 (0.3–9.0) |  | 4 | 4.9 (1.3–12.0) |
|  | Per participant | Any | 92 | 60 | 65.2 (54.6–74.9) | 94 | 65 | 69.1 (58.8–78.3) |
|  |  | Grade 3 |  | 6 | 6.5 (2.4–13.7) |  | 8 | 8.5 (3.7–16.1) |
| Myalgia | Visit 1 | Any | 92 | 59 | 64.1 (53.5–73.9) | 93 | 46 | 49.5 (38.9–60.0) |
|  |  | Grade 3 |  | 4 | 4.3 (1.2–10.8) |  | 1 | 1.1 (0.0–5.8) |
|  | Visit 2 | Any |  |  |  | 88 | 29 | 33.0 (23.3–43.8) |
|  |  | Grade 3 |  |  |  |  | 2 | 2.3 (0.3–8.0) |
|  | Visit 3 | Any | 78 | 41 | 52.6 (40.9–64.0) | 82 | 42 | 51.2 (39.9–62.4) |
|  |  | Grade 3 |  | 2 | 2.6 (0.3–9.0) |  | 4 | 4.9 (1.3–12.0) |
|  | Per participant | Any | 92 | 65 | 70.7 (60.2–79.7) | 94 | 65 | 69.1 (58.8–78.3) |
|  |  | Grade 3 |  | 5 | 5.4 (1.8–12.2) |  | 5 | 5.3 (1.7–12.0) |
| Arthralgia | Visit 1 | Any | 92 | 28 | 30.4 (21.3–40.9) | 93 | 20 | 21.5 (13.7–31.2) |
|  |  | Grade 3 |  | 2 | 2.2 (0.3–7.6) |  | 0 | 0.0 (0.0–3.9) |
|  | Visit 2 | Any |  |  |  | 88 | 18 | 20.5 (12.6–30.4) |
|  |  | Grade 3 |  |  |  |  | 2 | 2.3 (0.3–8.0) |
|  | Visit 3 | Any | 78 | 24 | 30.8 (20.8–42.2) | 82 | 23 | 28.0 (18.7–39.1) |
|  |  | Grade 3 |  | 3 | 3.8 (0.8–10.8) |  | 2 | 2.4 (0.3–8.5) |
|  | Per participant | Any | 92 | 38 | 41.3 (31.1–52.1) | 94 | 33 | 35.1 (25.5–45.6) |
|  |  | Grade 3 |  | 5 | 5.4 (1.8–12.2) |  | 4 | 4.3 (1.2–10.5) |
| Chills | Visit 1 | Any | 92 | 26 | 28.3 (19.4–38.6) | 93 | 15 | 16.1 (9.3–25.2) |
|  |  | Grade 3 |  | 1 | 1.1 (0.0–5.9) |  | 0 | 0.0 (0.0–3.9) |
|  | Visit 2 | Any |  |  |  | 88 | 17 | 19.3 (11.7–29.1) |
|  |  | Grade 3 |  |  |  |  | 1 | 1.1 (0.0–6.2) |
|  | Visit 3 | Any | 78 | 26 | 33.3 (23.1–44.9) | 82 | 21 | 25.6 (16.6–36.4) |
|  |  | Grade 3 |  | 3 | 3.8 (0.8–10.8) |  | 2 | 2.4 (0.3–8.5) |
|  | Per participant | Any | 92 | 38 | 41.3 (31.1–52.1) | 94 | 34 | 36.2 (26.5–46.7) |
|  |  | Grade 3 |  | 4 | 4.3 (1.2–10.8) |  | 3 | 3.2 (0.7–9.0) |
| GI symptoms | Visit 1 | Any | 92 | 21 | 22.8 (14.7–32.8) | 93 | 23 | 24.7 (16.4–34.8) |
|  |  | Grade 3 |  | 0 | 0.0 (0.0–3.9) |  | 1 | 1.1 (0.0–5.8) |
|  | Visit 2 | Any |  |  |  | 88 | 14 | 15.9 (9.0–25.2) |
|  |  | Grade 3 |  |  |  |  | 2 | 2.3 (0.3–8.0) |
|  | Visit 3 | Any | 78 | 8 | 10.3 (4.5–19.2) | 82 | 11 | 13.4 (6.9–22.7) |
|  |  | Grade 3 |  | 0 | 0.0 (0.0–4.6) |  | 0 | 0.0 (0.0–4.4) |
|  | Per participant | Any | 92 | 26 | 28.3 (19.4–38.6) | 94 | 33 | 35.1 (25.5–45.6) |
|  |  | Grade 3 |  | 0 | 0.0 (0.0–3.9) |  | 2 | 2.1 (0.3–7.5) |
| 60–69 YOA | | | | | | | | |
| Fever | Visit 1 | Any | 89 | 9 | 10.1 (4.7–18.3) | 91 | 4 | 4.4 (1.2–10.9) |
|  |  | Grade 3 |  | 4 | 4.5 (1.2–11.1) |  | 2 | 2.2 (0.3–7.7) |
|  | Visit 2 | Any |  |  |  | 86 | 2 | 2.3 (0.3–8.1) |
|  |  | Grade 3 |  |  |  |  | 1 | 1.2 (0.0–6.3) |
|  | Visit 3 | Any | 80 | 2 | 2.5 (0.3–8.7) | 89 | 7 | 7.9 (3.2–15.5) |
|  |  | Grade 3 |  | 0 | 0.0 (0.0–4.5) |  | 0 | 0.0 (0.0–4.1) |
|  | Per participant | Any | 90 | 11 | 12.2 (6.3–20.8) | 92 | 13 | 14.1 (7.7–23.0) |
|  |  | Grade 3 |  | 4 | 4.4 (1.2–11.0) |  | 3 | 3.3 (0.7–9.2) |
| Headache | Visit 1 | Any | 89 | 32 | 36.0 (26.1–46.8) | 91 | 29 | 31.9 (22.5–42.5) |
|  |  | Grade 3 |  | 0 | 0.0 (0.0–4.1) |  | 1 | 1.1 (0.0–6.0) |
|  | Visit 2 | Any |  |  |  | 86 | 19 | 22.1 (13.9–32.3) |
|  |  | Grade 3 |  |  |  |  | 0 | 0.0 (0.0–4.2) |
|  | Visit 3 | Any | 80 | 22 | 27.5 (18.1–38.6) | 89 | 31 | 34.8 (25.0–45.7) |
|  |  | Grade 3 |  | 0 | 0.0 (0.0–4.5) |  | 1 | 1.1 (0.0–6.1) |
|  | Per participant | Any | 90 | 40 | 44.4 (34.0–55.3) | 92 | 49 | 53.3 (42.6–63.7) |
|  |  | Grade 3 |  | 0 | 0.0 (0.0–4.0) |  | 2 | 2.2 (0.3–7.6) |
| Fatigue | Visit 1 | Any | 89 | 42 | 47.2 (36.5–58.1) | 91 | 37 | 40.7 (30.5–51.5) |
|  |  | Grade 3 |  | 2 | 2.2 (0.3–7.9) |  | 2 | 2.2 (0.3–7.7) |
|  | Visit 2 | Any |  |  |  | 86 | 24 | 27.9 (18.8–38.6) |
|  |  | Grade 3 |  |  |  |  | 1 | 1.2 (0.0–6.3) |
|  | Visit 3 | Any | 80 | 26 | 32.5 (22.4–43.9) | 89 | 45 | 50.6 (39.8–61.3) |
|  |  | Grade 3 |  | 0 | 0.0 (0.0–4.5) |  | 4 | 4.5 (1.2–11.1) |
|  | Per participant | Any | 90 | 48 | 53.3 (42.5–63.9) | 92 | 58 | 63.0 (52.3–72.9) |
|  |  | Grade 3 |  | 2 | 2.2 (0.3–7.8) |  | 7 | 7.6 (3.1–15.1) |
| Myalgia | Visit 1 | Any | 89 | 43 | 48.3 (37.6–59.2) | 91 | 37 | 40.7 (30.5–51.5) |
|  |  | Grade 3 |  | 0 | 0.0 (0.0–4.1) |  | 1 | 1.1 (0.0–6.0) |
|  | Visit 2 | Any |  |  |  | 86 | 24 | 27.9 (18.8–38.6) |
|  |  | Grade 3 |  |  |  |  | 2 | 2.3 (0.3–8.1) |
|  | Visit 3 | Any | 80 | 25 | 31.3 (21.3–42.6) | 89 | 38 | 42.7 (32.3–53.6) |
|  |  | Grade 3 |  | 0 | 0.0 (0.0–4.5) |  | 0 | 0.0 (0.0–4.1) |
|  | Per participant | Any | 90 | 52 | 57.8 (46.9–68.1) | 92 | 55 | 59.8 (49.0–69.9) |
|  |  | Grade 3 |  | 0 | 0.0 (0.0–4.0) |  | 3 | 3.3 (0.7–9.2) |
| Arthralgia | Visit 1 | Any | 89 | 18 | 20.2 (12.4–30.1) | 91 | 17 | 18.7 (11.3–28.2) |
|  |  | Grade 3 |  | 0 | 0.0 (0.0–4.1) |  | 0 | 0.0 (0.0–4.0) |
|  | Visit 2 | Any |  |  |  | 86 | 9 | 10.5 (4.9–18.9) |
|  |  | Grade 3 |  |  |  |  | 2 | 2.3 (0.3–8.1) |
|  | Visit 3 | Any | 80 | 14 | 17.5 (9.9–27.6) | 89 | 20 | 22.5 (14.3–32.6) |
|  |  | Grade 3 |  | 0 | 0.0 (0.0–4.5) |  | 0 | 0.0 (0.0–4.1) |
|  | Per participant | Any | 90 | 26 | 28.9 (19.8–39.4) | 92 | 31 | 33.7 (24.2–44.3) |
|  |  | Grade 3 |  | 0 | 0.0 (0.0–4.0) |  | 2 | 2.2 (0.3–7.6) |
| Chills | Visit 1 | Any | 89 | 24 | 27.0 (18.1–37.4) | 91 | 11 | 12.1 (6.2–20.6) |
|  |  | Grade 3 |  | 3 | 3.4 (0.7–9.5) |  | 0 | 0.0 (0.0–4.0) |
|  | Visit 2 | Any |  |  |  | 86 | 11 | 12.8 (6.6–21.7) |
|  |  | Grade 3 |  |  |  |  | 1 | 1.2 (0.0–6.3) |
|  | Visit 3 | Any | 80 | 15 | 18.8 (10.9–29.0) | 89 | 29 | 32.6 (23.0–43.3) |
|  |  | Grade 3 |  | 0 | 0.0 (0.0–4.5) |  | 2 | 2.2 (0.3–7.9) |
|  | Per participant | Any | 90 | 32 | 35.6 (25.7–46.3) | 92 | 39 | 42.4 (32.1–53.1) |
|  |  | Grade 3 |  | 3 | 3.3 (0.7–9.4) |  | 3 | 3.3 (0.7–9.2) |
| GI symptoms | Visit 1 | Any | 89 | 10 | 11.2 (5.5–19.7) | 91 | 11 | 12.1 (6.2–20.6) |
|  |  | Grade 3 |  | 0 | 0.0 (0.0–4.1) |  | 3 | 3.3 (0.7–9.3) |
|  | Visit 2 | Any |  |  |  | 86 | 10 | 11.6 (5.7–20.3) |
|  |  | Grade 3 |  |  |  |  | 0 | 0.0 (0.0–4.2) |
|  | Visit 3 | Any | 80 | 3 | 3.8 (0.8–10.6) | 89 | 13 | 14.6 (8.0–23.7) |
|  |  | Grade 3 |  | 0 | 0.0 (0.0–4.5) |  | 0 | 0.0 (0.0–4.1) |
|  | Per participant | Any | 90 | 11 | 12.2 (6.3–20.8) | 92 | 23 | 25.0 (16.6–35.1) |
|  |  | Grade 3 |  | 0 | 0.0 (0.0–4.0) |  | 3 | 3.3 (0.7–9.2) |
| ≥70 YOA | | | | | | | | |
| Fever | Visit 1 | Any | 77 | 6 | 7.8 (2.9–16.2) | 76 | 8 | 10.5 (4.7–19.7) |
|  |  | Grade 3 |  | 3 | 3.9 (0.8–11.0) |  | 2 | 2.6 (0.3–9.2) |
|  | Visit 2 | Any |  |  |  | 71 | 1 | 1.4 (0.0–7.6) |
|  |  | Grade 3 |  |  |  |  | 0 | 0.0 (0.0–5.1) |
|  | Visit 3 | Any | 72 | 0 | 0.0 (0.0–5.0) | 72 | 2 | 2.8 (0.3–9.7) |
|  |  | Grade 3 |  | 0 | 0.0 (0.0–5.0) |  | 1 | 1.4 (0.0–7.5) |
|  | Per participant | Any | 78 | 6 | 7.7 (2.9–16.0) | 78 | 10 | 12.8 (6.3–22.3) |
|  |  | Grade 3 |  | 3 | 3.8 (0.8–10.8) |  | 3 | 3.8 (0.8–10.8) |
| Headache | Visit 1 | Any | 77 | 21 | 27.3 (17.7–38.6) | 76 | 14 | 18.4 (10.5–29.0) |
|  |  | Grade 3 |  | 1 | 1.3 (0.0–7.0) |  | 1 | 1.3 (0.0–7.1) |
|  | Visit 2 | Any |  |  |  | 71 | 13 | 18.3 (10.1–29.3) |
|  |  | Grade 3 |  |  |  |  | 0 | 0.0 (0.0–5.1) |
|  | Visit 3 | Any | 72 | 15 | 20.8 (12.2–32.0) | 72 | 18 | 25.0 (15.5–36.6) |
|  |  | Grade 3 |  | 0 | 0.0 (0.0–5.0) |  | 0 | 0.0 (0.0–5.0) |
|  | Per participant | Any | 78 | 27 | 34.6 (24.2–46.2) | 78 | 32 | 41.0 (30.0–52.7) |
|  |  | Grade 3 |  | 1 | 1.3 (0.0–6.9) |  | 1 | 1.3 (0.0–6.9) |
| Fatigue | Visit 1 | Any | 77 | 32 | 41.6 (30.4–53.4) | 76 | 11 | 14.5 (7.5–24.4) |
|  |  | Grade 3 |  | 1 | 1.3 (0.0–7.0) |  | 0 | 0.0 (0.0–4.7) |
|  | Visit 2 | Any |  |  |  | 71 | 18 | 25.4 (15.8–37.1) |
|  |  | Grade 3 |  |  |  |  | 0 | 0.0 (0.0–5.1) |
|  | Visit 3 | Any | 72 | 25 | 34.7 (23.9–46.9) | 72 | 28 | 38.9 (27.6–51.1) |
|  |  | Grade 3 |  | 1 | 1.4 (0.0–7.5) |  | 1 | 1.4 (0.0–7.5) |
|  | Per participant | Any | 78 | 41 | 52.6 (40.9–64.0) | 78 | 33 | 42.3 (31.2–54.0) |
|  |  | Grade 3 |  | 2 | 2.6 (0.3–9.0) |  | 1 | 1.3 (0.0–6.9) |
| Myalgia | Visit 1 | Any | 77 | 32 | 41.6 (30.4–53.4) | 76 | 22 | 28.9 (19.1–40.5) |
|  |  | Grade 3 |  | 0 | 0.0 (0.0–4.7) |  | 1 | 1.3 (0.0–7.1) |
|  | Visit 2 | Any |  |  |  | 71 | 13 | 18.3 (10.1–29.3) |
|  |  | Grade 3 |  |  |  |  | 0 | 0.0 (0.0–5.1) |
|  | Visit 3 | Any | 72 | 19 | 26.4 (16.7–38.1) | 72 | 21 | 29.2 (19.0–41.1) |
|  |  | Grade 3 |  | 1 | 1.4 (0.0–7.5) |  | 0 | 0.0 (0.0–5.0) |
|  | Per participant | Any | 78 | 36 | 46.2 (34.8–57.8) | 78 | 34 | 43.6 (32.4–55.3) |
|  |  | Grade 3 |  | 1 | 1.3 (0.0–6.9) |  | 1 | 1.3 (0.0–6.9) |
| Arthralgia | Visit 1 | Any | 77 | 16 | 20.8 (12.4–31.5) | 76 | 5 | 6.6 (2.2–14.7) |
|  |  | Grade 3 |  | 0 | 0.0 (0.0–4.7) |  | 0 | 0.0 (0.0–4.7) |
|  | Visit 2 | Any |  |  |  | 71 | 4 | 5.6 (1.6–13.8) |
|  |  | Grade 3 |  |  |  |  | 0 | 0.0 (0.0–5.1) |
|  | Visit 3 | Any | 72 | 10 | 13.9 (6.9–24.1) | 72 | 8 | 11.1 (4.9–20.7) |
|  |  | Grade 3 |  | 1 | 1.4 (0.0–7.5) |  | 0 | 0.0 (0.0–5.0) |
|  | Per participant | Any | 78 | 21 | 26.9 (17.5–38.2) | 78 | 14 | 17.9 (10.2–28.3) |
|  |  | Grade 3 |  | 1 | 1.3 (0.0–6.9) |  | 0 | 0.0 (0.0–4.6) |
| Chills | Visit 1 | Any | 77 | 9 | 11.7 (5.5–21.0) | 76 | 7 | 9.2 (3.8–18.1) |
|  |  | Grade 3 |  | 0 | 0.0 (0.0–4.7) |  | 0 | 0.0 (0.0–4.7) |
|  | Visit 2 | Any |  |  |  | 71 | 4 | 5.6 (1.6–13.8) |
|  |  | Grade 3 |  |  |  |  | 1 | 1.4 (0.0–7.6) |
|  | Visit 3 | Any | 72 | 12 | 16.7 (8.9–27.3) | 72 | 13 | 18.1 (10.0–28.9) |
|  |  | Grade 3 |  | 0 | 0.0 (0.0–5.0) |  | 1 | 1.4 (0.0–7.5) |
|  | Per participant | Any | 78 | 15 | 19.2 (11.2–29.7) | 78 | 17 | 21.8 (13.2–32.6) |
|  |  | Grade 3 |  | 0 | 0.0 (0.0–4.6) |  | 2 | 2.6 (0.3–9.0) |
| GI symptoms | Visit 1 | Any | 77 | 10 | 13.0 (6.4–22.6) | 76 | 5 | 6.6 (2.2–14.7) |
|  |  | Grade 3 |  | 2 | 2.6 (0.3–9.1) |  | 0 | 0.0 (0.0–4.7) |
|  | Visit 2 | Any |  |  |  | 71 | 4 | 5.6 (1.6–13.8) |
|  |  | Grade 3 |  |  |  |  | 0 | 0.0 (0.0–5.1) |
|  | Visit 3 | Any | 72 | 10 | 13.9 (6.9–24.1) | 72 | 6 | 8.3 (3.1–17.3) |
|  |  | Grade 3 |  | 0 | 0.0 (0.0–5.0) |  | 0 | 0.0 (0.0–5.0) |
|  | Per participant | Any | 78 | 17 | 21.8 (13.2–32.6) | 78 | 11 | 14.1 (7.3–23.8) |
|  |  | Grade 3 |  | 2 | 2.6 (0.3–9.0) |  | 0 | 0.0 (0.0–4.6) |

**Notes:** Fever was defined as a temperature ≥38.0°C. Grade 3 AEs were defined as fever >39.0°C, and headache, fatigue, myalgia, arthralgia, chills, GI symptoms that prevented normal everyday activities. ^a^95% CIs were based on the Clopper-Pearson CI method.

**Abbreviations:** AE, adverse event; Co-Ad group, co-administration of RZV dose 1 with adjuvanted RSVPreF3; RZV, adjuvanted recombinant herpes zoster subunit vaccine; adjuvanted RSVPreF3, adjuvanted respiratory syncytial virus prefusion F protein-based vaccine; Control group, sequential administration of RZV dose 1 and adjuvanted RSVPreF3; N, number of participants with completed eDiary card; eDiary, electronic diary; n (%), number (percentage) of participants in each group with completed eDiary cards presenting at least one type of symptom; CI, confidence interval; YOA, years of age; GI, gastrointestinal.

### Supplementary Table 7: Proportion of participants with unsolicited AEs and SAEs, by age group (exposed set)

| **AEs** | **Co-Ad group** | | |  | **Control group** | | |
| --- | --- | --- | --- | --- | --- | --- | --- |
|  | **50–59 YOA**  **N=92** | **60–69 YOA**  **N=93** | **≥70 YOA**  **N=80** |  | **50–59 YOA**  **N=94** | **60–69 YOA**  **N=93** | **≥70 YOA**  **N=78** |
| **Unsolicited AEs^a^, n (%)** |  |  |  |  |  |  |  |
| Any unsolicited AEs | 24 (26.1) | 20 (21.5) | 18 (22.5) |  | 28 (29.8) | 28 (30.1) | 24 (30.8) |
| Grade 3 unsolicited AEs | 1 (1.1) | 0 (0.0) | 1 (1.3) |  | 1 (1.1) | 1 (1.1) | 1 (1.3) |
| **Any SAEs^b^, n (%)** | 2 (2.2) | 5 (5.4) | 6 (7.5) |  | 2 (2.1) | 2 (2.2) | 2 (2.6) |

**Notes:** ^a^Unsolicited AEs were recorded within 30 days post-any vaccination. ^b^SAEs were recorded up to study end.

**Abbreviations:** AE, adverse event; SAE, serious AE; Co-Ad group, co-administration of RZV dose 1 with adjuvanted RSVPreF3; RZV, adjuvanted recombinant herpes zoster subunit vaccine; adjuvanted RSVPreF3, adjuvanted respiratory syncytial virus prefusion F protein-based vaccine; Control group, sequential administration of RZV dose 1 and adjuvanted RSVPreF3; YOA, years of age; N, total number of participants; n (%), number (percentage) of participants in each group reporting the AE.
